# Cardiopulmonary and metabolic responses during a 2-day CPET in myalgic encephalomyelitis/chronic fatigue syndrome: translating reduced oxygen consumption to impairment status to treatment considerations

**DOI:** 10.1186/s12967-024-05410-5

**Published:** 2024-07-05

**Authors:** Betsy Keller, Candace N. Receno, Carl J. Franconi, Sebastian Harenberg, Jared Stevens, Xiangling Mao, Staci R. Stevens, Geoff Moore, Susan Levine, John Chia, Dikoma Shungu, Maureen R. Hanson

**Affiliations:** 1 Department of Exercise Science and Athletic Training, Ithaca College, Ithaca, NY 14850 USA; 2 Department of Molecular Biology and Genetics, Cornell University, Ithaca, NY 14853 USA; 3 Department of Human Kinetics, St. Francis Xavier University, Antigonish, NS B2G 2W5 Canada; 4 Department of Radiology, Weill Cornell Medicine, New York, NY 10065 USA; 5 Workwell Foundation, Ripon, CA 95366 USA; 6 Susan Levine, MD Clinical Practice, New York, NY 10021 USA; 7 ID Med, Torrance, CA 90505 USA

**Keywords:** ME/CFS, Chronic fatigue syndrome, Functional impairment, Fatigue, Post exertional malaise, Cardiopulmonary exercise test, Autonomic dysfunction, Two-day CPET

## Abstract

**Background:**

Post-exertional malaise (PEM), the hallmark symptom of myalgic encephalomyelitis/chronic fatigue syndrome (ME/CFS), represents a constellation of abnormal responses to physical, cognitive, and/or emotional exertion including profound fatigue, cognitive dysfunction, and exertion intolerance, among numerous other maladies. Two sequential cardiopulmonary exercise tests (2-d CPET) provide objective evidence of abnormal responses to exertion in ME/CFS but validated only in studies with small sample sizes. Further, translation of results to impairment status and approaches to symptom reduction are lacking.

**Methods:**

Participants with ME/CFS (Canadian Criteria; n = 84) and sedentary controls (CTL; n = 71) completed two CPETs on a cycle ergometer separated by 24 h. Two-way repeated measures ANOVA compared CPET measures at rest, ventilatory/anaerobic threshold (VAT), and peak effort between phenotypes and CPETs. Intraclass correlations described stability of CPET measures across tests, and relevant objective CPET data indicated impairment status. A subset of case–control pairs (n = 55) matched for aerobic capacity, age, and sex, were also analyzed.

**Results:**

Unlike CTL, ME/CFS failed to reproduce CPET-1 measures during CPET-2 with significant declines at peak exertion in work, exercise time, $\dot{\text{V}}$e, $\dot{\text{V}}$O_2_, $\dot{\text{V}}$CO_2_, $\dot{\text{V}}$_T_, HR, O_2_pulse, DBP, and RPP. Likewise, CPET-2 declines were observed at VAT for $\dot{\text{V}}$e/$\dot{\text{V}}$CO_2_, PetCO_2,_ O_2_pulse, work, $\dot{\text{V}}$O_2_ and SBP. Perception of effort (RPE) exceeded maximum effort criteria for ME/CFS and CTL on both CPETs. Results were similar in matched pairs. Intraclass correlations revealed greater stability in CPET variables across test days in CTL compared to ME/CFS owing to CPET-2 declines in ME/CFS. Lastly, CPET-2 data signaled more severe impairment status for ME/CFS compared to CPET-1.

**Conclusions:**

Presently, this is the largest 2-d CPET study of ME/CFS to substantiate impaired recovery in ME/CFS following an exertional stressor. Abnormal post-exertional CPET responses persisted compared to CTL matched for aerobic capacity, indicating that fitness level does not predispose to exertion intolerance in ME/CFS. Moreover, contributions to exertion intolerance in ME/CFS by disrupted cardiac, pulmonary, and metabolic factors implicates autonomic nervous system dysregulation of blood flow and oxygen delivery for energy metabolism. The observable declines in post-exertional energy metabolism translate notably to a worsening of impairment status. Treatment considerations to address tangible reductions in physiological function are proffered.

*Trial registration number:* ClinicalTrials.gov, retrospectively registered, ID# NCT04026425, date of registration: 2019-07-17.

## Background

Profound and protracted fatigue that is not alleviated by rest, unrefreshing sleep, brain fog, dysregulation of heart rate, blood pressure and ventilation, and symptom exacerbation following physical, cognitive and/or emotional stressors, only begins to describe the daily burden of one who is ill with Myalgic Encephalomyelitis/Chronic Fatigue Syndrome (ME/CFS) [1]. In 2015, the *Institute of Medicine* (IOM: now, *National Academy of Medicine*) produced a comprehensive review of studies of ME/CFS [1]. One objective of the report was to better inform physicians about the multi-symptom neuroimmune disease and to aid in making a diagnosis by providing a relatively simple set of diagnostic criteria. Ideally, this would reduce the number of visits to specialists in search of a diagnosis [2] and shorten time to diagnosis to less than the typical 2 to 5 + years (https://solvecfs.org/wp-content/uploads/2014/01/IOM_RoadtoDiagnosisSurveyReport.pdf). In 2019, it was estimated that 3.4 million Americans [3] and 67 million people worldwide [4] suffered with ME/CFS. With no known cause, cure, or definitive diagnostic biomarker, an estimated 90% with ME/CFS remain undiagnosed [1]. Due to the debilitating nature of ME/CFS, those with moderate to severe symptoms are unable to maintain pre-illness employment, social life, and require assistance with basic and instrumental activities of daily living [5]. Both people with ME/CFS and family members/care-givers are highly negatively impacted financially as well as socially [6].

Despite a lack of biomarker for ME/CFS, there is robust evidence of disrupted immune function [7–12], neural activity [13–15], and metabolic function [16–18]. Still, the antecedent of ME/CFS remains to be determined. Nevertheless, clinical evidence of impaired energy production and recovery following an exertional stressor has been observed repeatedly in ME/CFS during a two-day cardiopulmonary exercise test protocol (2-d CPET) [19–28].

Production of energy relies on the coordinated functions of the cardiovascular, pulmonary, and musculoskeletal systems to provide oxygenated blood to central and peripheral structures for energy metabolism. Regulation by the autonomic nervous system (ANS) oversees these processes to ensure that oxygen demands are met where needed to produce energy for work. Insufficient oxygen delivery due directly to ANS dysfunction, and/or indirectly via dysregulated feedback loop mechanisms that provide input from the periphery, can impair energy production due to reduced delivery of oxygenated blood. Thus, objective evidence from CPET associated with ANS-regulated processes (e.g., hemodynamics, ventilation, etc.) may signal dysregulation of these systems to provide blood flow and oxygen delivery for energy production to do work. A previous study of a single CPET with healthy controls and ME/CFS matched for peak aerobic capacity ($\dot{\text{V}}$O_2_peak) identified predominantly ventilatory-related measures that distinguish ME/CFS [29, 30]. However, impaired recovery following exertion, measured with a subsequent CPET, may better elucidate the impact of post-exertional symptom exacerbation or post-exertional malaise (PEM). Further, a second CPET to assess the magnitude of de-integrated metabolism provides for the objective quantification of impaired recovery on capacity to do work. To date, the present study is the largest to assess baseline (test 1) and recovery (test 2) energy metabolism in ME/CFS compared to healthy, inactive controls.

## Aims

The primary objectives of this study were to assess; (1) baseline aerobic capacity (peak oxygen consumption) as a measure of energy producing capacity, (2) ability to recover normally following a standardized exercise stressor, (3) degree of impairment associated with baseline aerobic capacity and impaired recovery, and (4) exercise responses in ME/CFS compared to sedentary controls in cardiovascular, pulmonary, and musculoskeletal contributors to aerobic capacity and energy production. A secondary purpose was to compare a subset of ME/CFS and sedentary controls matched for sex, age, and peak oxygen consumption to determine if the effects of these factors align with previous reports of cardiopulmonary function in ME/CFS [29, 30].

## Methods

This was a multi-site initiative and part of the Collaborative Research Center (CRC) for the study of ME/CFS at Cornell University funded by the NIH under a U54 cooperative agreement. Participants were enrolled at three sites following a two-stage screening process by expert clinicians to assess for eligibility. Participants completed a 2-d CPET to assess baseline and recovery indices, including (1) physiologic, metabolic, and cardiopulmonary measures, and for the broader CRC initiatives, (2) biospecimens collected before and after both exercise tests, (3) illness characteristics obtained via questionnaires and psychometric instruments, (4) symptom severity before, during and after exercise tests, and (5) quantification by accelerometry of baseline and post-exercise tests physical activity levels. Data discussed herein is limited to selected psychometric instruments, and physiologic, metabolic, and cardiopulmonary measures from the 2-day CPET.

### Recruitment: participant screening

Participants accepted into the protocol completed testing at one of three sites, including Ithaca College (Ithaca, NY), Weill Cornell Medicine (New York, NY), and an infectious disease clinic, ID Med (Torrance, CA). Institutional Review Board approval was obtained from Ithaca College (for Ithaca College and ID Med; IRB# 1017-12D)) and from Weill Cornell Medicine (protocol# 1708018518). Participants were recruited in respective regions of each test site (upstate NY, LA, NYC) throughout a 4-year period during which data collection took place from 2018 to 2022. Recruitment included local/regional efforts at the three test sites via electronic and hard copy advertisements at ME/CFS specialist clinics (ID Med in LA, Medical Office of Susan Levine in NYC), via postings to various ME/CFS advocacy network websites (e.g., MEAction), and on ClinicalTrials.gov. Screening of potential participants who responded to our advertisements involved two phases beginning with a screening phone call, and if deemed eligible, an invitation to schedule for the second phase medical screening by either John Chia, MD (LA), Susan Levine, MD (NYC), or Geoff Moore, MD (upstate NY). Oral informed consent was obtained for the screening phone call and written informed consent was obtained for the medical screening and subsequent protocol. Interested potential participants completed the screening phone call to assess preliminary eligibility regarding exclusion/inclusion criteria and physical activity level (for sedentary controls; CTL), to explain the protocol requirements, and to address any questions. This screening phase identified participants who initially did not meet inclusion criteria, for example, due to a high level of physical activity, or were unwilling or unable to follow the protocol. Further, this filtered the number of potential participants who qualified for medical screening by a study physician. Those who met all inclusion and no exclusion criteria, and CTL who also endorsed a low/no recreational/vocational physical activity level, were invited to schedule for the medical screening. Recruitment, screening, and testing were interrupted in March 2020 due to the SARS CoV-2 pandemic. This forced a pause in the study due, in part, to the overwhelming demands on physicians to treat pandemic patients, and to ensure the safety and welfare of participants and researchers. The on-going pandemic-related demands varied regionally such that all test sites were not able to resume testing at the same time. The second phase medical screening included administration of the Modified Mini Screen [31], confirmation of prescribed medications, screening physical exam (HEENT, cardiovascular, musculoskeletal, etc.), details of current health concerns, endorsement of ME/CFS symptoms and compliance with Canadian Consensus Criteria [32], urine analysis for recreational drugs and pregnancy status, and screening blood sample (TSH, CBC, CMP, HbA1C) to rule out, for example, thyroid dysfunction, type II diabetes, and other metabolic disorders. Any candidate who was found to have a previously unknown medical diagnosis and/or inadequately medically managed diagnosis that could contribute to fatigue (e.g., type 2 diabetes) was referred to their doctor with an invitation to return once the diagnosis was medically stable. Those who passed both screening phases and consented to participate in the protocol were also required to provide a physician clearance from their personal medical provider.

*Inclusion criteria* were ages 18–70 y, diagnosis of ME/CFS (for ME/CFS), endorsement of low physical activity level (for CTL), willingness to follow protocol as detailed in Informed Consent, willingness to stop taking supplements/probiotics two weeks prior to CPETs and pain and/or stimulants 2 days prior to CPETs.

*Exclusion criteria* were pregnant or breast feeding, current smoker, current infectious illness, musculoskeletal problem that would preclude ability to perform CPET on a cycle ergometer, excessive alcohol consumption, diabetic using insulin, or unstable medical problem to contraindicate performing a CPET.

### Multi-site coordination

A clinical co-director (BK) for the Cornell CRC study met with the site physicians, research coordinators and study principal investigators to orient to the clinical testing operating procedures. Thereafter, BK met with the test site coordinators (XM, JS) to assure compliance with the 2-d CPET protocol regarding participant recruitment, screening, CPET procedures, physical activity monitoring, CPET recovery follow-up, conveyance of biosamples, and secure transfer of study data between test sites. In addition, BK met with trained exercise technicians at each site to prepare for and practice the test protocol, confirm calibration and long-term quality control and assurance with biocalibration of metabolic measurement systems [33], as well as electronically-braked cycle ergometers and blood pressure cuffs. Metabolic measurement systems at each site underwent routine annual maintenance as recommended by the manufacturer (MGC Diagnostics Corp., MN).

### Baseline questionnaires

Screening questionnaires included a cardiovascular risk stratification screening [34], physical activity history screening, and a medical/health/lifestyle/demographics status questionnaire. Psychometric instruments included Bell Activity Scale [35] and Specific Symptom Survey [36, 37]. ME/CFS-only were provided pre-CPET preparation guidelines and strategies to assist recovery following CPETs. Participants were also provided detailed travel directions to the buildings/clinics and parking to minimize walking/energy expenditure upon arrival.

### Cardiopulmonary exercise test (CPET)

The CPET protocol was an incremental, ramping workload on an accommodating-resistance electronically-braked cycle ergometer. Following 3 min of seated rest on the ergometer, the exercise protocol began at 0 Watts (W) with an incremental rise in work intensity of 15 W throughout each minute of exercise at the rate of 5W over 20 s. Participants completed two maximum effort CPETs separated by 24 h.

#### Pre-CPET preparation

Participants were asked to refrain from ingestion of food at least 2 h prior to CPET, caffeine and carbonated beverages 4 h prior to CPET, and alcohol and cannabis 24 h prior to CPET. Current smokers or those who quit less than one year earlier were not admitted into the protocol. Also, participants were asked to refrain from exercise at least 24 h prior to scheduled CPET. ME/CFS participants were asked to arrive for testing in a rested, ‘baseline’ state (no symptom exacerbation), which may have required pre-rest for multiple days prior to the scheduled CPET. Additionally, participants agreed to stop use of vitamins, minerals, and pro/pre-biotics two weeks prior, and pain medications two days prior to CPET-1.

#### CPET

Testing environment for CPETs was consistent varying between 20 and 24 °C and 40–60% humidity. Upon arrival to the testing room, a participant completed forms to verify compliance with pre-CPET preparation requirements and a pre-CPET Specific Symptom Survey. Height (cm) was measured using a stadiometer prior to CPET 1, and body weight (kg) was measured using a balance beam scale before each CPET. The participant rested quietly, supine, with arms at side and legs/knees supported at ~ 35° angle for 5 min, after which resting electrocardiogram (ECG; 12-lead) and blood pressure (BP) were measured. The participant was then oriented to the dyspnea scale (1–4), pain scale (1–4), and Rating of Perceived Exertion scale (RPE, 6–20; [38]). Resting ratings of pain and dyspnea were obtained. The participant was then seated on the cycle ergometer and seat height was determined so that knee angle was ~ 165° with the foot resting level on the pedal at bottom dead center. The participant was instrumented with a finger-worn pulse oximeter (SpO_2_) and a facemask/breathing valve, followed by a facemask leak challenge to check mask fit. Thereafter, expired respiratory gases were collected during 3 min of seated rest with BP and SpO_2_ recorded at minute 2.

#### Exercise protocol

Use of a low-intensity ramp cycle protocol allows for minimal overall exercise time for the ME/CFS participant because the first minute of cycling (0 to 15W) requires low effort and serves as a warm-up. This is particularly important to do when testing people with ME/CFS because a customary 3-min unloaded cycling warm-up can require sufficient energy demand of those with ME/CFS to elicit the gas exchange threshold (ventilatory/anaerobic threshold; VAT) during the warm-up phase. If this were to occur, the typical physiological responses to incremental exercise would be obscured due to the premature onset of VAT during the warm-up [39]. To avoid this problem, after 3 min of seated rest during which expired gases were collected, the participant began the cycle protocol directly with a low-effort ramping of work intensity during the first minute from 0 to 15W. Workload increments continued at 15W per minute until volitional exhaustion, the prescribed pedal rate of 50–80 rpm could not be maintained, the participant requested to stop, or the exercise technician deemed it unsafe for the participant to continue. ECG was monitored continuously during seated rest, exercise, and throughout recovery. Measures of blood pressure, SpO_2_ by pulse oximetry, and ratings of RPE, dyspnea, and pain were obtained beginning at minute 1 of cycling and every 2 min thereafter. Verbal encouragement was administered similarly and consistently across CPET-1 and CPET-2 to offer support for a maximum effort.

#### Post-test recovery

Upon test termination, workload was reduced to 0 W and unloaded cycling was continued for a one-minute cool-down during which BP was obtained. Reason for test termination, peak RPE, pain and dyspnea ratings were confirmed with the participant as well. At the end of recovery minute 1, the participant was moved to a plinth positioned immediately adjacent to the cycle ergometer for supine recovery with legs elevated as described earlier. Elevated leg position is helpful to aid recovery particularly for most ME/CFS who experience orthostatic intolerance. Monitoring of expired gases and ECG continued throughout recovery. Blood pressure and SpO_2_ were recorded at minutes 1, 3, 5, and every 2 min thereafter until heart rate returned to within 20 bpm of pre-test resting level and blood pressure was close to pre-test resting level [40]. Seated then standing posture were allowed based on signs/symptoms of orthostatic regulation. Water was offered once the participant was able to sit.

#### CPET-2

CPET-2 was completed 24 h (± 2 h) following CPET-1 and followed the same procedures described above. Other than to verify valid and accurate data acquisition and post-test calibration of the metabolic measurement system, CPET-1 data was not scrutinized prior to conducting CPET-2 to minimize risk of participant priming during CPET-2.

### Metabolic and cardiopulmonary measurements

#### Maximum effort

The validity of a maximum effort CPET as an indicator of aerobic (functional or maximum) capacity requires the participant to achieve maximum exertion during the test unless maximum exertion is otherwise limited by a sign or symptom that precludes maximum effort or places the participant at undue risk. Three indices of exertion were measured during CPET to assess for maximum effort; (1) respiratory exchange ratio (RER) ≥ 1.10, (2) attainment of heart rate greater than or equal to 85% of age-predicted maximum heart rate, and (3) RPE ≥ 17/20. Assessment of VO_2_ plateau was not considered for the assessment of maximum effort due to the unreliability of this criterion in certain populations [41–43]. Further, reports of ventilatory dysfunction in ME/CFS [44] could affect attainment of a VO_2_ plateau resulting in a misinterpretation of maximum effort. Attainment of two of three criteria is considered acceptable to determine that maximum effort was achieved in healthy individuals. However, exertion in any individual may be limited by a sign or symptom that precludes maximum effort or places the participant at undue risk [40].

#### Gas exchange measures

Measurements of expired gases were made breath-by-breath using indirect calorimetry via open-circuit spirometry with a metabolic measurement system (MGC Diagnostics, MN, Ultima System PFX Cardio2, model# 800860-011) to determine oxygen consumption ($\dot{\text{V}}$O_2_), carbon dioxide production ($\dot{\text{V}}$CO_2_), ventilation volume ($\dot{\text{V}}$e), and related ventilatory indices. Expired ventilation measures were obtained via a pneumotach attached to an oronasal facemask (preVent®, MGC Diagnostics, MN), and expired gases were sampled from a line coupled to the pneumotach. Use of a facemask improves comfort and reduces risk of coughing/gagging, particularly for those with ME/CFS, and produces similar results to maximum effort tests with a mouthpiece [45]. Calibration of gas sensors was completed before and after CPET using standard calibration gases of verified concentrations (12% O_2_, 5% CO_2_, balance N_2_) and reference gas concentrations (21% O_2_, balance N_2_). Calibration of the pneumotach was achieved using a three-liter calibration syringe at five different flow rates. VAT, a common analog for anaerobic threshold, was determined by the metabolic cart with the V-slope algorithm [46] using the mid 5 of 7 breath-by-breath values, as well as visual assessment by well-trained investigators (BK, JS) of the non-linear increase in Ve, the non-linear increase in $\dot{\text{V}}$CO_2_, and the nadir followed by a positive inflection of $\dot{\text{V}}$e/$\dot{\text{V}}$O_2_ and PetO_2_.

Additional cardiopulmonary performance variables were derived from measures of gas exchange, hemodynamic function, and work to provide insight into the interrelatedness of cardiovascular, pulmonary, and metabolic factors to produce energy for work. These derived variables are described more fully in the Results section.

### Data management

Data (CPET, questionnaires) were inspected for completeness first by respective test site coordinators (JS, BK, XM). Exercise test data from each test site were securely transmitted to the study office at Ithaca College for data management and processing. Raw gas exchange data was transformed from breath-by-breath to 20-s averages to reduce variability to an acceptable level and identify data outliers, missing data, and/or sampling inconsistencies by the metabolic measurement system. Transformation of data to 20-s averages began at the start of 3-min seated rest and re-commenced at the start of exercise until end of recovery. When workload was reduced at test termination prior to the end of a 20-s interval, the final exercise VO_2_ and associated measures were calculated from breath-by-breath data using the last six seconds of exercise data.

### VO_2_peak matched pairs

A subset of ME/CFS-CTL participants were matched on baseline aerobic capacity ($\dot{\text{V}}$O_2_peak) to assess whether differences in metabolic measures between phenotypes were due to differences in baseline aerobic capacity. Such differences, described previously in a study using a single CPET protocol, reported that matching ME/CFS and CTL for $\dot{\text{V}}$O_2_peak reduced many of the differences between phenotypes in the total sample such that only differences in ventilatory function persisted among the matched-pairs [30]. While it was unclear how the matched-pairs were identified in this study, it was reported that 11 of 99 matched-pairs included males matched to females. Due to sex differences in cardiovascular factors that contribute to $\dot{\text{V}}$O_2_peak (cardiac size, arterial diameter, blood volume, 2,3 DPG, hemoglobin concentration, hematocrit, etc.) [47–49], pairs analyzed in the present study were matched first by sex, then by age, and lastly by baseline (CPET 1) $\dot{\text{V}}$O_2_peak. No males were matched with females. A total of 55 matched pairs were identified for the subset analysis.

### Data analysis

Power analysis: The target comparison to determine the appropriate sample size for the present study was the difference in peak VO_2_ between ME/CFS and CTL. Previous research reported effect sizes as large as 0.66 (Hedges g) [30]. To ensure sufficient power, a more conservative estimated effect size (0.45) was chosen. Based on a power analysis with α at 0.05 and power at 0.80 (using G Power 2), the recommended sample size was 158 participants (i.e., 79 participants per group).

Continuous variables were summarized in means and standard deviations. Categorical variables were aggregated in means and percentages. Before the calculation of parametric statistics was conducted, normality of continuous variables was assessed. As none of the variables showed abnormality of data, parametric statistics were deemed appropriate. Participant characteristics were compared between phenotype groups by sex and between groups for the total sample using independent samples t-test with Cohen’s d effect sizes or χ^2^ tests with Cramer’s V effect sizes (for categorical variables), and 95% confidence intervals. Measures from CPET were compared between groups (ME/CFS, CTL) and within CPETs (CPET-1, CPET-2) using multiple repeated measures two-way ANOVAs. Post hoc adjustment of significant group by time interactions were done using the Holm-Bonferroni method [50] and Cohen’s d effect. Stability of CPET variables over tests was assessed using intraclass correlation coefficients with a 95% confidence interval. All analyses were conducted using JASP (version 0.17.2) with a set α level of 0.05.

## Results

### Participants

Participants accepted and consented to the protocol included 171 individuals (81 CTL, 90 ME/CFS) across the three test sites. Statistical analysis of the 2-day CPET data required successful completion of both CPETs for participant data to be included in the analysis. Data excluded from the final statistical analyses included participants who; 1) did not satisfy the maximum effort criteria explained previously for CPET-1 or CPET-2 (4 CTL), 2) were missing either test 1 or test 2 due to technical problems (2 CTL, 4 ME/CFS), 3) were controls with baseline (CPET-1) VO_2_peak 2.5 + standard deviations higher than the sample mean for sex/phenotype (4 CTL), 4) had VO_2_peak with ‘superior’ category rating for age/sex (1 ME/CFS), and 5) misrepresented sex classification on intake and screening responses (1 ME/CFS). The final sample size in the analysis included 155 participants (71 CTL, 84 ME/CFS). ME/CFS participants who met maximum effort criteria during test 1 but did not meet criteria during test 2 due to failure to achieve heart rate and/or RER criteria were not excluded from analysis. ME/CFS who did not meet heart rate or RER criteria during test 2 but did meet RPE criteria (perception of maximum effort) are emblematic of post-exertion symptom exacerbation that contributes to exertion intolerance. For example, chronotropic incompetence and/or other symptoms of dysautonomia that emerge following exertion (CPET-1) may preclude the ability to satisfy the heart rate or RER criteria during test 2.

### Participant characteristics

Participant characteristics for the total sample and matched pair subset appear in Table 1a, b, respectively. For the full sample size, females comprised 75%, 72%, and 74%, respectively, of the ME/CFS, CTL, and total sample, which is consistent with the prevalence of ME/CFS reported in the general population[3]. In general, the ME/CFS group was comprised of more Caucasians (*p* = 0.001), spent more hours per day in bed (*p* < 0.001), had a higher incidence of unrefreshing sleep (p < 0.001), and rated significantly lower on the Bell Activity Scale[35] (*p* < 0.001). Groups were similar in height and weight, while ME/CFS was about 4 years older (*p* = 0.04). Female ME/CFS were about 6 years older (*p* = 0.02) with slightly lower body mass index (BMI; *p* = 0.02) compared to female CTL. Likewise, BMI was slightly lower in the total ME/CFS group compared to CTL (*p* = 0.04).

**Table 1:** **a** Descriptive and baseline characteristics data for ME/CFS and controls. **b** Descriptive and baseline characteristics data for VO_2_peak-matched pairs

a									
	ME/CFS			Controls			*p-*level, ES (CI)*		
	Females	Males	Total	Females	Males	Total	Females	Males	Total
N (%total)	63 (75%)	21	84	51 (72%)	20	71	114 (74%)	41	155
Age (y)	47.2 (11.3)	45.8 (10.5)	46.9 (11.1)	41.8 (13.7)	45.4 (12.5)	42.8 (13.4)	p = .02* d = −.44 (−.81-−.06)	p = .92 d = −.03 (−.64-.58)	p = .04* d = −.33 (−.65-−.01)
Height (m)	1.65 (0.06)	1.77 (0.06)	1.68 (0.08)	1.64 (0.05)	1.80 (0.09)	1.68 (0.10)	p = .15 d = −.27 (−.64-.10)	p = .28 d = .34 (−.28-.96)	p = 1.0 d = 0.0 (−.32-.32)
Weight (kg)	71.6 (15.8)	87.2 (12.9)	75.5 (16.5)	76.9 (16.6)	89.4 (20.7)	80.4 (18.6)	p = .08 d = .33 (−.04-.70)	p = .69 d = .13 (−.49-.74)	p = .08 d = .16 (−.04-.60)
BMI (kg/m^2^)	26.2 (5.4)	27.9 (4.1)	26.6 (5.2)	28.7 (5.8)	27.4 (4.4)	28.3 (5.5)	p = .02* d = .45 (.08-.82)	p = .74 d = −.11 (−.72-.51)	p = .04* d = .33 (.01-.65)
HR rest (bpm)	82.3 (12.2)	76.0 (8.7)	80.8 (11.7)	81.5 (8.9)	78.5 (11.0)	80.7 (9.5)	p = .69 d = −.08 (−.45-.29)	p = .42 d = .25 (−.36-.87)	p = .96 d = −.01 (−.32-.31)
SBP rest (mmHg)	119.8 (19.3)	122.5 (14.9)	120.5 (18.3)	113.0 (14.4)	121.8 (11.7)	115.5 (14.2)	p = .04* d = −.40 (−.77-.02)	p = .86 d = −.05 (−.67-.56)	p = .06 d = −.31 (−.62-.01)
DBP rest (mmHg)	79.1 (10.8)	79.5 (9.8)	79.2 (10.5)	73.4 (9.4)	79.7 (7.7)	75.1 (9.3)	p = .003** d = −.56 (−.94-.19)	p = .95 d = .02 (−.59-.63)	p = .01** d = −.41 (−.73-−.09)
Baseline $\dot{\text{V}}$O_2_peak (ml^.^kg^−1.^min^−1^)	20.0 (4.7)	23.3 (6.2)	20.8 (5.3)	21.7 (4.8)	25.4 (5.5)	22.7 (5.3)	p = .08 d = .34 (−.03-.71)	p = .26 d = .36 (−.26-.97)	p = .03* d = .35 (.03-.67)
Race %Caucasian (n)	92% (58)	90% (19)	92% (77)	71% (36)	75% (15)	72% (51)	p = .030*, V = .28	p = .19, V = .21	p = .001**, V = .26
Education %college (n)	76% (48)	65% (13)^†^	73% (61)	62% (31)	80% (16)	67% (47)	p = .10, V = .15	p = .20, V = .17	p = .39, V = .07
#Hours in bed/day	10.0 (1.8)	9.3 (1.4)	9.8 (1.7)	8.3 (2.6)	8.0 (1.1)	8.2 (2.3)	p < .001**, d = −.74 (−.11-−^.^35)	P = .001**, d = −1.08 (−1.73-−.41)	p < .001**, d = −.78 (−1.11-−.45)
%Unrefreshing sleep (n)	90% (56)	90% (19)	90% (75)	24% (12)	5% (1)	18% (13)	p < .001** V = .68	p < .001** V = .86	p < .001** V = .73
Bell Activity Scale	33.9 (11.0)	39.3 (15.4)	35.2 (12.4)	94.7 (9.1)	96.0 (6.0)	95.1 (8.3)	p < .001**, d = 5.96 (5.09-6.83)	P < .001**, d = 4.82 (3.58-6.01)	p < .001**, d = 5.59 (4.88-6.28)

b									
N (75% female pairs)	41	14	55	41	14	55			
Age (y)	45.1 (12.4)	48.1 (9.3)	45.9 (11.6)	45.1 (12.4)	45.9 (10.0)	45.3 (11.8)	p = .98 d = .00 (–.44-.43)	p = .56 d = −.22 (−.9-–.52)	p = .79 d = −.05 (−.42-.32)
Height (m)	1.66 (.07)	1.77 (.06)	1.69 (.08)	1.64 (.05)	1.82 (.09)	1.68 (.10)	p = .17 d = −.31 (−.74-.13)	p = .10 d = .66 (−.11-1.41)	p = .96 d = −.01 (−.38-.37)
Weight (kg)	71.0 (15.8)	87.4 (12.9)	75.1 (16.7)	79.4 (16.8)	97.0 (19.9)	83.9 (19.1)	p = .02* d = .52 (.08-.96)	p = .14 d = .58 (−.19-1.32)	p = .01** d = .49 (.11-.87)
BMI (kg/m^2^)	25.8 (5.2)	27.7 (3.2)	26.3 (4.9)	29.5 (5.8)	28.9 (4.1)	29.4 (5.4)	p = .003** d = .69 (.24-1.13)	p = .39 d = .33 (−.42-1.07)	p = .002** d = .61 (.23-.99)
HR rest (bpm)	82.9 (13.3)	75.4 (9.0)	80.9 (12.7)	80.6 (9.1)	82.1 (8.7)	81.0 (8.9)	p = .38 d = −.20 (−.63-.24)	p = .05* d = .77 (−.01-1.53)	p = .98 d = −.01 (−.37-.38)
SBP rest (mmHg)	118.6 (17.8)	121.1 (16.7)	119.3 (17.4)	115.1 (15.0)	121.7 (12.2)	116.8 (14.6)	p = .33 d = −.22 (−.65-.22)	p = .92 d = .04 (−.70-.78)	p = .42 d = −.16 (−.53-.22)
DBP rest (mmHg)	79.0 (10.1)	79.7 (10.6)	79.1 (10.1)	74.1 (9.8)	79.1 (8.1)	75.4 (9.6)	p = .03* d = −.49 (−.92-–.05)	p = .87 d = −.06 (−.80 -.68)	p = .05* d = −.38 (−.76- .00)
Baseline $\dot{\text{V}}$O_2_peak (ml^.^kg^−1.^min^−1^)	21.1 (4.4)	23.5 (3.9)	21.7 (4.4)	21.2 (4.5)	22.8 (4.2)	21.6 (4.4)	p = .88 d = .04 (−.40-.47)	p = .65 d = −.17 (−.91-.57)	p = .−94 d = −.02 (−.39-.36)
Race %Caucasian (n)	88% (36)	86% (12)	80% (44)	78% (32)	86% (12)	87% (48)	p = .24, V = .13	p = 1.00, V = .00	p = .30, V = .10
Education %college degree (n)	83% (34)	64% (9)	69% (37)	65% (26)	79% (11)	78% (43)	p = .07, V = .21	p = .40, V = .16	p = .25, V = .11
#Hours in bed/day	9.9 (1.7)	9.4 (1.4)	9.8 (1.6)	8.4 (2.9)	7.9 (1.1)	8.0 (2.5)	P = .009** d = −.60 (−1.10-−.15)	P = .005** d = −1.16 (−1.95-−.35)	p < .001** d = −.67 (−1.06-−.29)
%Unrefreshing sleep (n)	93% (38)	100% (14)	95% (52)	27% (11)	0% (0)	20% (11)	p < .001**, V = .67	p < .001**, V = 1.00	p < .001**, V = .75
Bell Activity Scale	34.5 (10.6)	38.2 (15.6)	35.5 (12.0)	95.3 (8.2)	95.0 (6.5)	95.2 (7.7)	p < .001** d = 6.41 (5.32-7.50)	p < .001** d = 4.74 (3.25-6.21)	p < .001** d = 5.90 (5.02-6.77)

*BMI* body mass index, *HR* heart rate, *SBP* systolic pressure, *DBP* diastolic pressure, Baseline VO_2_peak (test 1); ^†^N = 20

*p ≤ 0.05, **p ≤ 0.01

Regarding baseline physiological measures, $\dot{\text{V}}$O_2_peak was not significantly different between phenotypes by sex but was 9% higher in the total group of CTL (*p* = 0.03). Interestingly, both baseline (resting) SBP and DBP were comparatively higher in ME/CFS females (*p* = 0.04; *p* = 0.003) and DBP was higher in all ME/CFS (*p* = 0.01). The differences between phenotypes were largely influenced by the higher proportion of ME/CFS females versus males in the total sample.

For ME/CFS-CTL pairs matched for sex, age, and peak $\dot{\text{V}}$O_2_ (Table 1b, N = 55), as expected, there were no differences for age within pairs. CTL females weighed more (*p* = 0.02) with higher BMI (*p* = 0.003). Body weight (*p* = 0.01) and BMI (*p* = 0.002) were also higher in CTL of the total group and matched-pairs. Unlike the full sample, there was no difference in race between matched-pairs. However, as with the full sample, ME/CFS spent more hours per day in bed (*p* < 0.001), had a higher incidence of unrefreshing sleep (*p* < 0.001), and rated significantly lower on the Bell Activity Scale (*p* < 0.001). Once matched for sex, age and $\dot{\text{V}}$O_2_peak, differences in resting SBP disappeared, whereas resting DBP remained significantly higher in both ME/CFS females (*p* = 0.03) and all ME/CFS (*p* = 0.05).

### CPET measures—peak effort

**Fig. 1:**
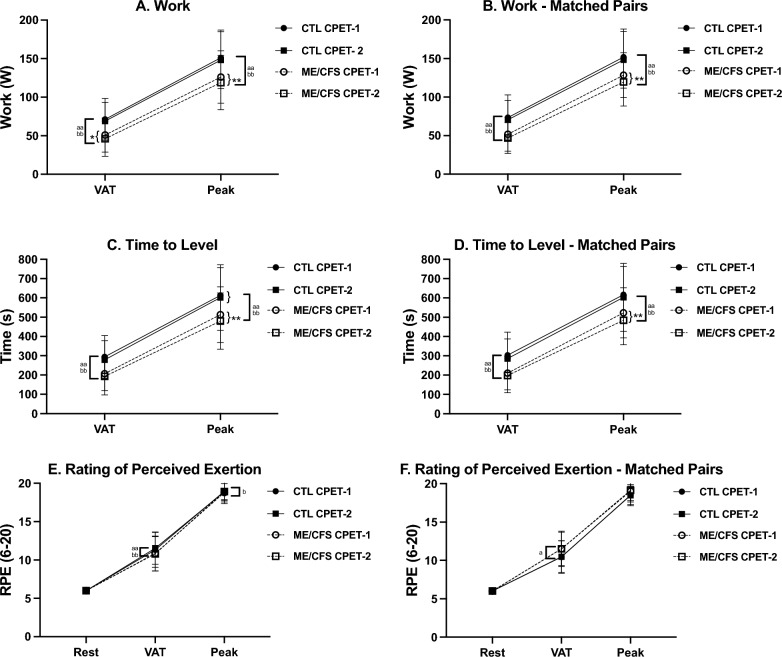
Work-related variables for CPET-1 and CPET-2 for ME/CFS and controls. Changes in measures related to cardiopulmonary exercise tests (CPET) across ventilatory/anaerobic threshold (VAT) and peak exercise in ME/CFS (n = 84) and controls (CTL; n = 71) and VO_2_peak-matched pairs (n = 55). Measures were collected during cardiopulmonary exercise tests (CPET) across day 1 (CPET-1) and day 2 (CPET-2) and include work (**A**, **B**), time to level (**C**, **D**), and rating of perceived exertion (RPE; **E**, **F**). Data were analyzed using 2-way repeated measures ANOVA and are presented as mean ± SD. *denotes p ≤ 0.05 between CPETs, **p ≤ 0.01 between CPETs, ^a^*p* ≤ 0.05 between groups for CPET-1, ^aa^*p* ≤ 0.01 between groups for CPET-1, ^b^*p* ≤ 0.05 between groups for CPET-2, ^bb^*p* ≤ 0.01 between groups for CPET-2

**Fig. 2:**
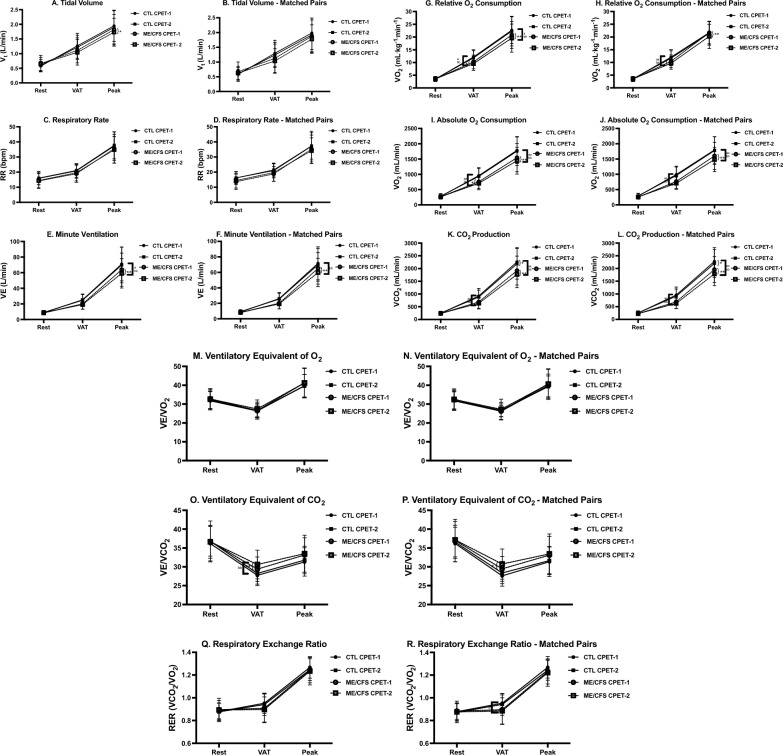
Ventilatory variables for CPET-1 and CPET-2 for ME/CFS and controls. Changes in measures related to ventilatory function across rest, ventilatory/anaerobic threshold (VAT), and peak exercise in ME/CFS (n = 84) and controls (CTL; n = 71) and VO_2_peak-matched pairs (n = 55). Measures were collected during cardiopulmonary exercise tests (CPET) across day 1 (CPET-1) and day 2 (CPET-2) and include tidal volume (TV; **A**, **B**), respiratory rate (RR; **C**, **D**), minute ventilation (VE; **E**, **F**), relative (**G**, **H**) and absolute (**I**, **J**) O_2_ consumption, CO_2_ production (**K**, **L**), ventilatory equivalents of O_2_ (VE/VO_2_; **M**, **N**) and CO_2_ (VE/VCO_2_; **O**, **P**), and respiratory exchange ratio (RER; **Q**, **R**). Data were analyzed using 2-way repeated measures ANOVA and are presented as mean ± SD. *denotes *p* ≤ 0.05 between CPETs, **p ≤ 0.01 between CPETs, ^a^*p* ≤ 0.05 between groups for CPET-1, ^aa^*p* ≤ 0.01 between groups for CPET-1, ^b^*p* ≤ 0.05 between groups for CPET-2, ^bb^*p* ≤ 0.01 between groups for CPET-2

**Fig. 3:**
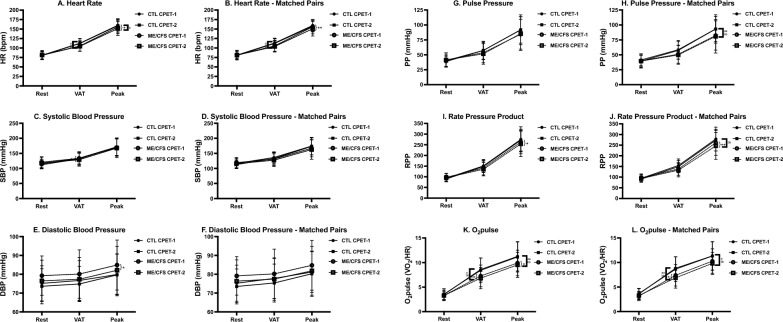
Hemodynamic variables for CPET-1 and CPET-2 for ME/CFS and controls. Changes in measures related to hemodynamic function across rest, ventilatory/anaerobic threshold (VAT), and peak exercise in ME/CFS (n = 84) and controls (CTL; n = 71) and VO_2_peak-matched pairs (n = 55). Measures were collected during cardiopulmonary exercise tests (CPET) across day 1 (CPET-1) and day 2 (CPET-2) and include heart rate (HR; **A**, **B**), systolic blood pressure (SBP; **C**, **D**), diastolic blood pressure (DBP; **E**, **F**), pulse pressure (PP; **G**, **H**), rate pressure product (RPP; **I**, **J**), and O_2_pulse (**K**, **L**). Data were analyzed using 2-way repeated measures ANOVA and are presented as mean ± SD. *denotes *p* ≤ 0.05 between CPETs, **p ≤ 0.01 between CPETs, ^a^*p* ≤ 0.05 between groups for CPET-1, ^aa^*p* ≤ 0.01 between groups for CPET-1, ^b^*p* ≤ 0.05 between groups for CPET-2, ^bb^*p* ≤ 0.01 between groups for CPET-2

Cardiopulmonary measures at peak effort appear in Table 2 and Figs. 1, 2, 3and4 for the total sample and the pairs matched for sex, age and $\dot{\text{V}}$O_2_peak. A comparison of CPET-1 to CPET-2 for the total sample revealed numerous highly significant differences (*p* ≤ 0.01) albeit with small to moderate effect sizes in ME/CFS at peak effort for Work, Time to level (TTL), $\dot{\text{V}}$e, $\dot{\text{V}}$O_2_, $\dot{\text{V}}$CO_2_, and significant differences (*p* ≤ 0.05) in $\dot{\text{V}}$_T_, heart rate (HR), O_2_pulse, DBP, and rate-pressure product (RPP). In contrast for CTL, significant differences between CPETs were observed only for $\dot{\text{V}}$CO_2_ (*p* ≤ 0.05).

**Table 2:** Cardiopulmonary measures at peak VO_2_ for ME/CFS and controls for CPET-1 and CPET-2

	Total sample						VO_2_peak-matched pairs					
	ME/CFS (n = 84)			Controls (n = 71)			ME/CFS (n = 55)			Controls (n = 55)		
	CPET-1	CPET-2	ES (CI)	CPET-1	CPET-2	ES (CI)	CPET-1	CPET-2	ES (CI)	CPET-1	CPET-2	ES (CI)
Work (W)	126.2 (34.2)	119.3 (35.2)	.23^**^ (.04-.42)	151.0^aa^ (36.0)	148.0^bb^ (37.0)	.10 (−.11-.31)	130.1 (31.5)	121.9 (32.5)	.30^**^ (.03-.58)	150.9^aa^ (37.0)	147.1^bb^ (37.3)	.13 (−.13-.39)
Time to level (sec)	513.4 (144.7)	481.3 (146.4)	.25^**^ (.04-.47)	612.8^aa^ (144.2)	601.9 ^bb^ (170.1)	.09 (−.15-.32)	529.6 (138.1)	488.4 (124.3)	.32^**^ (.02-.63)	612.4^aa^ (148.4)	598.7^bb^ (177.9)	.11 (−.18-.39)
RER	1.24 (.11)	1.23 (.12)	.08 (−.26-.43)	1.27 (.10)	1.25 (.10)	.21 (−.17-.59)	1.23 (.11)	1.23 (.12)	.08 (−.36-.51)	1.27 (.10)	1.24 (.09)	.24 (−.21-.68)
$\dot{\text{V}}$ e (L/min)	64.0 (21.4)	59.4 (18.8)	.36^**^ (.14-.58)	71.1^a^ (21.7)	70.0^bb^ (23.0)	.09 (−.14-.32)	66.0 (20.8)	60.9 (18.3)	.40^**^ (.12-.69)	71.2 (21.5)	69.5^bb^ (21.2)	.16 (−.12-.39)
RR (breaths/min)	35.6 (9.6)	35.0 (8.7)	.09 (−.19-.38)	37.3 (9.5)	37.8 (9.0)	−.07 (−.38-.24)	35.4 (9.3)	34.4 (8.2)	.16 (−.19-.51)	37.1 (9.9)	37.4 (8.6)	−.05 (−.40-.30)
$\dot{\text{V}}$_T_ (L/min)	1.82 (.53)	1.73 (.48)	.22^*^ (−.02-.45)	1.95 (.53)	1.90 (.57)	.13 (−.12-.38)	1.89 (.55)	1.80 (.50)	.22 (−.06-.50)	1.98 (.56)	1.91 (.57)	.16 (−.12-.44)
$\dot{\text{V}}$ O_2_ (ml^.^kg^−1.^min^−1^)	20.8 (5.3)	19.7 (5.5)	.33^**^ (.12-.54)	22.7^a^ (5.3)	22.4^bb^ (5.8)	.10 (−.12-.32)	21.7 (4.4)	20.4 (4.6)	.42^**^ (.13-.70)	21.6 (4.4)	21.3 (4.7)	.10 (−.15-.34)
$\dot{\text{V}}$ O_2_ (ml/min)	1547.4 (464.7)	1451.8 (437.3)	.32^**^ (.13-.51)	1786.5^aa^ (434.9)	1760.8^bb^ (476.7)	.09 (−.11-.29)	1611.1 (439.7)	1504.6 (394.7)	.37^**^ (.12-.61)	1789.3^aa^ (436.5)	1764.4^bb^ (459.9)	.09 (−.13-.30)
$\dot{\text{V}}$ CO_2_ (ml/min)	1916.8 (572.3)	1774.3 (523.1)	.39^**^ (.18-.61)	2261.2^aa^ (562.5)	2187.2^bb^ (601.8)	.21^*^ (−.02-.43)	1973.3 (515.3)	1820.6 (469.9)	.45^**^ (.16-.74)	2264.4^aa^ (548.1)	2183.3^bb^ (553.0)	.24^*^ (−.03-.50)
$\dot{\text{V}}$ e/$\dot{\text{V}}$ O_2_	41.3 (7.7)	41.3 (8.0)	.00 (−.31-.32)	39.7 (6.0)	39.6 (6.1)	.02 (−.32-.37)	41.0 (8.0)	40.8 (8.2)	.04 (−.35-.43)	39.8 (6.0)	39.4 (5.7)	.06 (−.33-.46)
$\dot{\text{V}}$ e/$\dot{\text{V}}$ CO_2_	33.2 (4.5)	33.5 (4.9)	−.08 (−.35-.20)	31.3 (3.8)	31.8 (3.7)	−.12 (−.42-.18)	33.2 (5.0)	33.5 (5.2)	−.05 (−.39-.28)	31.3 (3.9)	31.7 (3.7)	−.10 (−.44-.24)
HR (bpm)	155.7 (17.7)	151.8 (18.0)	.27^*^ (.00-.55)	159.9 (17.5)	159.0^b^ (16.6)	.07 (−.22-.36)	156.9 (18.8)	151.7 (18.6)	.36^**^ (.01-.72)	159.6 (15.8)	157.9 (14.9)	.12 (−.21-.46)
%pred HR_max_	90.0 (0.1)	87.7 (0.1)	.24^*^ (.03-.45)	90.4 (0.1)	89.8 (0.1)	.07 (−.15-.28)	90.1 (10.4)	87.2 (10.5)	.32^**^ (.07-.56)	91.4 (7.8)	90.4 (7.2)	.11 (−.13-.34)
O_2_pulse ($\dot{\text{V}}$ O_2_/HR)	9.9 (2.6)	9.5 (2.6)	.16^*^ (−.03-.35)	11.3^aa^ (3.0)	11.2^bb^ (3.1)	.06 (−.15-.26)	10.2 (2.5)	9.9 (2.3)	.16 (−.06-.39)	11.3^a^ (2.9)	11.3^bb^ (2.9)	.04 (−.18-.25)
SBP (mmHg)	169.5 (28.7)	167.8 (31.7)	.08 (−.15-.30)	171.9 (29.8)	171.4 (27.7)	.02 (−.22-.27)	167.5 (28.3)	164.2 (32.8)	.15 (−.14-.43)	175.3 (28.3)	173.7 (27.8)	.07 (−.21-.35)
DBP (mmHg)	84.9 (13.3)	81.8 (12.5)	.28^*^ (.00-.56)	79.9 (10.8)	79.7 (11.2)	.02 (−.27-.31)	84.8 (13.2)	81.9 (12.9)	.26 (−.08-.59)	81.5 (10.8)	80.7 (11.7)	.08 (−.25-.40)
PP (mmHg)	84.7 (24.9)	86.0 (28.2)	−.07 (−.36-.21)	92.0 (24.9)	91.7 (22.1)	.02 (−.29-.33)	82.8 (24.1)	82.4 (28.6)	.02 (−.34-.39)	93.8^aa^ (23.4)	93.0^bb^ (21.3)	.04 (−.33-.41)
RPP	263.6 (55.2)	254.7 (59.6)	.23^*^ (−.02-.49)	275.4 (58.8)	271.9 (51.0)	.09 (−.18-.36)	263.5 (57.9)	250.6 (63.3)	.34^**^ (.02-.66)	280.2 (54.4)	274.1^bb^ (49.8)	.16 (−.15-.47)
PETO_2_ (mmHg)	118.0 (5.8)	117.8 (5.5)	.02 (−.28-.32)	114.6^a^ (4.4)	114.5^b^ (4.6)	.02 (−.30-.34)	118.1 (6.3)	117.7 (6.0)	.07 (−.29-.42)	114.5^aa^ (4.1)	114.3^b^ (4.2)	.04 (−.31-.39)
PETCO_2_ (mmHg)	34.1 (4.8)	33.7 (4.6)	.10 (−.14-.33)	36.0 (4.8)	35.4 (4.1)	.14 (−.11-.39)	34.2 (5.3)	33.9 (5.1)	.07 (−.21-.36)	36.1 (4.9)	35.5 (4.2)	.14 (−.15-.43)
RPE (6–20)	19.2 (1.1)	19.3 (1.3)	−.07 (−.37-.23)	18.5 (1.3)	18.5^b^ (1.2)	.02 (−.30-.35)	19.1 (1.2)	19.2 (1.5)	−.06 (−.42-.30)	18.5 (1.4)	18.5 (1.2)	.01 (−.35-.37)

Time to level = time to peak exertion, *RER* respiratory exchange ratio, $\dot{\text{V}}$ e minute ventilation, *RR* respiratory rate, $\dot{\text{V}}$_T_ tidal volume, $\dot{\text{V}}$ e/$\dot{\text{V}}$ O_2_ ventilatory equivalent of oxygen, $\dot{\text{V}}$ e/$\dot{\text{V}}$ CO_2_ ratio of ventilatory equivalent of carbon dioxide, *HR* heart rate, *%pred HR*_*max*_ actual peak HR/(220-age), *SBP* systolic pressure, *DBP* diastolic pressure, *PP* pulse pressure, *RPP* rate pressure product; *PETO*_*2*_ pressure end tidal O_2_, *PETCO*_*2*_ pressure end tidal CO_2_, *RPE* rating of perceived exertion (6–20)

*p ≤ 0.05, **p ≤ 0.01 between CPETs, ^a^*p* ≤ 0.05, ^aa^*p* ≤ 0.01 between groups for CPET-1, ^b^*p* ≤ 0.05, ^bb^*p* ≤ 0.01 between groups for CPET-2.

**Fig. 4:**
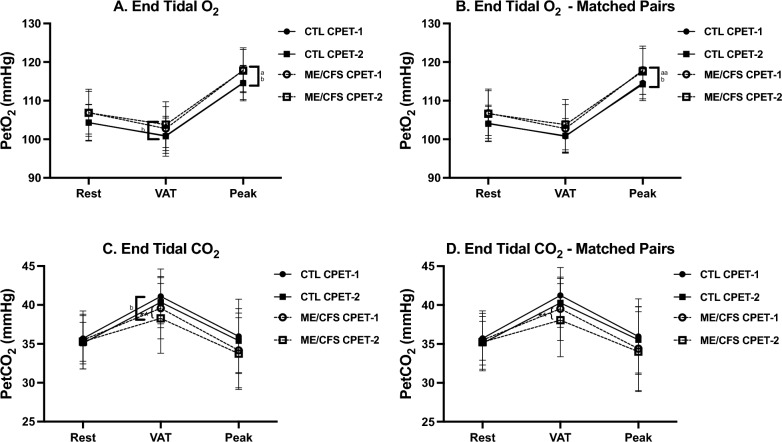
End tidal gas measures throughout CPET-1 and CPET-2 for ME/CFS and controls. Changes in measures related to end tidal gases across rest, ventilatory/anaerobic threshold (VAT), and peak exercise in ME/CFS (n = 84) and controls (CTL; n = 71) and VO_2_peak-matched pairs (n = 55). Measures were colleged during cardiopulmonary exercise tests (CPET) across day 1 (CPET-1) and day 2 (CPET-2) and include end tidal O_2_ (**A**, **B**) and end tidal CO_2_ (**C**, **D**). Data were analyzed using 2-way repeated measures ANOVA and are presented as mean ± SD. *denotes *p* ≤ 0.05 between CPETs, **p ≤ 0.01 between CPETs, ^a^*p* ≤ 0.05 between groups for CPET-1, ^aa^*p* ≤ 0.01 between groups for CPET-1, ^b^*p* ≤ 0.05 between groups for CPET-2, ^bb^*p* ≤ 0.01 between groups for CPET-2

In the matched-pairs sample, a comparison of CPET-1 to CPET-2 for ME/CFS revealed persistent declines in 8 of 11 CPET measures in the total ME/CFS sample discussed above, with $\dot{\text{V}}$_T_, O_2_pulse, and DBP no longer significantly different between tests. However, two cardiovascular measures (HR, RPP) that decreased on CPET-2 (*p* ≤ 0.05) in the total sample were also lower on CPET-2 (*p* ≤ 0.01) in ME/CFS in the matched-pairs. Thus, the post-exertional deterioration of CPET measures during CPET-2 further corroborates the impact of PEM on oxygen delivery and energy production in ME/CFS. In contrast, only one measure ($\dot{\text{V}}$CO_2_) for CTL in the matched-pairs decreased from CPET-1 to CPET-2 (*p* ≤ 0.01), further supporting the well-established high reliability and consistency of peak CPET measures [51], and consistent with the comparatively high intraclass correlation coefficients (ICC) for CTL discussed below (Table 5).

For the total sample, CTL was higher than ME/CFS during CPET-1 for Work, TTL, $\dot{\text{V}}$O_2 (ml/min)_, $\dot{\text{V}}$CO_2_ (*p* ≤ 0.01), and $\dot{\text{V}}$e, $\dot{\text{V}}$O_2 (ml.kg-1.min-1)_ and PetO_2_ (*p* ≤ 0.05). Despite screening as ‘low-active’ controls, baseline $\dot{\text{V}}$O_2_peak for CTL was 8% higher compared to ME/CFS, which likely contributed to higher measures of Work, TTL, and gas exchange. Differences between phenotypes for CPET-2 were also evident but occurred primarily due to a decline in CPET-2 measures in ME/CFS rather than an increase in CTL measures. Persistent higher measures in CTL remained for Work, TTL, $\dot{\text{V}}$e, $\dot{\text{V}}$O_2 (ml.kg-1.min-1)_, $\dot{\text{V}}$O_2 (ml/min)_, O_2_pulse, $\dot{\text{V}}$CO_2_ (*p* ≤ 0.01), and HR, PetO_2_, and lower RPE (*p* ≤ 0.05).

For matched-pairs, significant differences between phenotypes for CPET-1 were the same as those in the total sample, with two exceptions. First, there was no difference for peak $\dot{\text{V}}$O_2 (ml.kg-1.min-1)_ because phenotypes were matched based on CPET-1 peak $\dot{\text{V}}$O_2_. Second, unlike the total sample, CTL had a significantly higher pulse pressure (PP, 13%) (*p* ≤ 0.01) compared to ME/CFS matched for peak $\dot{\text{V}}$O_2_. As with the total sample, differences between phenotypes for CPET-2 occurred primarily due to declines in CPET-2 measures in ME/CFS and were largely consistent with phenotype differences for CPET-1. Notably, however, was a significant 6% decline in peak $\dot{\text{V}}$O_2 (ml.kg-1.min-1)_ on CPET-2 (*p* ≤ 0.01) in ME/CFS with no change in CTL. As in CPET-1, PP for CTL in the matched pairs was also 13% higher than ME/CFS during CPET-2 (*p* ≤ 0.01). Lastly, although differences between phenotypes for SBP did not achieve statistical significance, a trending decline from CPET-1 to CPET-2 was evident for ME/CFS with an overall lower SBP compared to CTL. The collective decrease in SBP and HR for ME/CFS on CPET-2 contributed to a significant 5% reduction in RPP (*p* ≤ 0.01).

### CPET measures—ventilatory/anaerobic threshold

Table 3 includes the cardiopulmonary measures at VAT for the total sample and the matched pairs. A comparison of CPET-1 to CPET-2 for the total sample of ME/CFS elucidated three significant differences (*p* ≤ 0.01) at VAT for $\dot{\text{V}}$e/$\dot{\text{V}}$CO_2_, PetCO_2,_ O_2_pulse, and four significant differences (*p* ≤ 0.05) including Work, VO_2_ (absolute & relative to body weight), and SBP with small to moderate effect sizes. In contrast, there were no significant differences for CTL between tests with comparatively smaller effect sizes for all variables. In the matched-pairs sample, a comparison of CPET-1 to CPET-2 for ME/CFS revealed persistent declines in 4 of 7 CPET measures in the total ME/CFS sample. Undoubtedly, the smaller size of the matched pairs sample affected some comparisons as the mean values for Work, for example, were similar to the total sample, but did not differ significantly for the matched pairs. The remaining differences were consistent with the total sample of ME/CFS with significant differences for PetCO_2_ (*p* ≤ 0.01), VO_2 (ml.kg-1.min-1)_, $\dot{\text{V}}$e/$\dot{\text{V}}$CO_2 slope_, and O_2_pulse (*p* ≤ 0.05). The post-exertional deterioration of CPET measures during CPET-2 demonstrates the deleterious impact of post-exertional malaise (PEM) on energy production in ME/CFS. As with the total sample, there were no differences in any measures at VAT from CPET-1 to CPET-2 in the matched-pairs CTL, providing evidence of greater stability of CPET measures at VAT in CTL. This was also observed in the comparatively higher ICCs for CTL (Table 6).

**Table 3:** Cardiopulmonary measures at ventilatory/anaerobic threshold (VAT) for ME/CFS and Controls for CPET-1 and CPET-2

	Total Sample						VO_2_peak-matched pairs					
	ME/CFS (n = 84)			Controls (n = 71)			ME/CFS (n = 55)			Controls (n = 55)		
	CPET-1	CPET-2	ES (CI)	CPET-1	CPET-2	ES (CI)	CPET-1	CPET-2	ES (CI)	CPET-1	CPET-2	ES (CI)
Work (W)	51.2 (22.3)	46.4 (22.8)	.16^*^ (−.03-.35)	71.3^aa^ (27.2)	68.9^bb^ (24.4)	.08 (–﻿.12-.29)	51.8 (22.6)	47.7 (21.7)	.14 (−.12-.39)	73.5^aa^ (29.1)	70.0^bb^ (25.0)	.12 (−.14-.37)
Time to level (sec)	208.3 (88.1)	193.9 (96.5)	.11 (−.10-.33)	294.3^aa^ (110.7)	279.3^bb^ (98.9)	.12 (−.11-.35)	211.0 (89.0)	201.7 (94.6)	.07 (−.21-.36)	304.2^aa^ (118.6)	283.7^bb^ (101.8)	.16 (−.13-.45)
RER	.91 (.13)	.90 (.11)	.10 (−.25-.44)	.95 (.09)	.94 (.10)	.11 (−.26-.49)	.89 (.13)	.88 (.12)	.11 (−.33-.55)	.96^a^ (.09)	.94 (.10)	.13 (−.31-.57)
$\dot{\text{V}}$e (L/min)	20.1 (6.8)	19.3 (5.9)	.06 (−.15-.27)	25.3 (7.4)	24.7 (7.3)	.05 (−.18-.27)	20.2 (7.3)	19.5 (6.4)	.05 (−.20-.31)	26.1 (7.8)	25.5 (7.5)	.05 (−.20-.31)
RR (breaths/min)	19.0 (5.7)	19.7 (5.3)	−.11 (−.39-.18)	20.8 (4.9)	21.1 (4.5)	−.04 (−.35-.27)	18.7 (4.9)	19.7 (5.7)	−.16 (−.51-.19)	21.1 (4.9)	21.5 (4.5)	−.06 (−.40-.29)
$\dot{\text{V}}$_T_(L/min)	1.11 (.43)	1.04 (.43)	.17 (−.07-.40)	1.27 (.42)	1.22 (.41)	.13 (−.13-.38)	1.13 (.48)	1.05 (.42)	.20 (− .08-.48)	1.30 (.44)	1.23 (.43)	.13 (−.14-.41)
$\dot{\text{V}}$O_2_ (ml^.^kg^−1.^min^−1^)	10.3 (2.7)	9.6 (2.6)	.19^*^ (−.01-.39)	12.7^a^ (2.9)	11.8^bb^ (2.8)	.10 (−.12-.31)	10.4 (2.5)	9.7 (2.4)	.21^*^ (−.04-.47)	12.0^aa^ (2.9)	11.6^bb^ (2.9)	.13 (−.12-.38)
$\dot{\text{V}}$O_2_ (ml/min)	758.0 (222.1)	709.4 (214.9)	.16^*^ (−.02-.35)	960.0^aa^ (260.2)	934.5^bb^ (264.6)	.09 (−.11-.29)	766.7 (224.5)	719.1 (208.1)	.16 (−.06-.38)	989.7^aa^ (271.0)	958.7^bb^ (276.7)	.11 (−.11-.32)
$\dot{\text{V}}$CO_2_ (ml/min)	689.8 (251.8)	637.6 (224.4)	.15 (−.06-.35)	922.8^aa^ (296.7)	882.4^bb^ (274.6)	.11 (−.11-.33)	690.4 (260.7)	636.8 (214.1)	.16 (−.10-.41)	956.7^aa^ (311.9)	907.4^bb^ (284.6)	.14 (−.11-.40)
$\dot{\text{V}}$ e/$\dot{\text{V}}$O_2_	26.6 (4.6)	27.5 (4.8)	−.17 (−.49-.15)	26.4 (3.1)	26.6 (3.7)	−.04 (−.38-.31)	26.2 (4.7)	27.2 (5.4)	−.18 (−.57-.22)	26.4 (3.1)	26.7 (3.4)	−.06 (−.46-.33)
$\dot{\text{V}}$e/$\dot{\text{V}}$CO_2_	29.5 (3.2)	30.7 (3.7)	−.31^**^ (−.60-−.03)	27.7 (2.7)	28.3^bb^ (2.8)	−.13 (−.43-.17)	29.5 (3.2)	30.8 (3.9)	−.31^*^ (–.﻿66-.04)	27.6 (2.7)	28.4 (2.8)	−.18 (−.52-.16)
HR (bpm)	105.5 (13.7)	104.6 (13.4)	.06 (−.21-.33)	112.6^a^ (12.6)	112.3^b^ (12.1)	.02 (−.27-.31)	105.1 (14.6)	104.1 (14.6)	.07 (−.26-.41)	113.7^a^ (13.0)	112.4^b^ (11.8)	.09 (−.25-.42)
%peak $\dot{\text{V}}$O_2_	50.0 (10.0)	50.0 (10.0)	.00 (−.25-.29)	54.2^a^ (10.0)	.53.8^b^ (10.0)	.05 (−.23-.33)	48.3 (8.6)	48.5 (10.0)	–.02 (−.34-.31)	55.7^aa^ (8.3)	54.7^bb^ (8.4)	.11 (−.22-.44)
O_2_pulse ($\dot{\text{V}}$O_2_/HR)	7.3 (2.2)	6.8 (2.2)	.20^**^ (.00-.39)	8.6^aa^ (2.4)	8.4^bb^ (2.5)	.08 (−.13-.29)	7.4 (2.2)	7.0 (2.1)	.19^*^ (−.03-.42)	8.8^aa^ (2.4)	8.6^bb^ (2.5)	.07 (−.15-.29)
SBP (mmHg)	133.6 (23.0)	128.6 (21.6)	.22^*^ (−.01-.45)	134.0 (19.0)	132.2 (19.9)	.08 (−.17-.32)	131.7 (21.2)	127.5 (21.3)	.19 (−.10-.47)	137.0 (18.7)	133.6 (20.1)	.15 (−.14-.44)
DBP (mmHg)	79.6 (12.7)	76.9 (11.3)	.24 (−.03-.52)	76.7 (10.5)	74.9 (9.4)	.17 (−.13-.46)	79.8 (13.0)	77.2 (11.1)	.23 (−.10-.57)	78.3 (10.6)	75.8 (9.9)	.22 (−.12-.55)
PP (mmHg)	54.0 (17.1)	51.7 (17.5)	.12 (−.16-.41)	57.3 (15.3)	57.3 (14.8)	.00 (−.31-.31)	51.9 (15.3)	50.3 (15.7)	.09 (−.28-.46)	58.7 (15.5)	57.8 (14.5)	.05 (−.32-.42)
RPP	141.5 (33.1)	134.7 (31.1)	.18 (−.08-.43)	151.3 (30.2)	148.5 (28.8)	.07 (−.20-.34)	138.7 (30.1)	133.1 (31.6)	.15 (−.16-.45)	156.3 (30.8)	150.3 (28.4)	.16 (−.15-.46)
PETO_2_ (mmHg)	102.8 (5.7)	103.9 (5.9)	−.20 (−.50-.09)	100.9 (4.6)	100.8^b^ (5.2)	.03 (−.29-.35)	102.8 (6.1)	103.9 (6.4)	−.20 (−.56-.16)	100.9 (4.2)	100.9 (4.5)	.00 (−.36-.35)
PETCO_2_ (mmHg)	39.5 (3.8)	38.1 (4.3)	.34^**^ (.10-.59)	41.1 (3.5)	40.4^b^ (3.3)	.19 (−.07-.44)	39.5 (43.9)	37.9 (4.4)	.39^**^ (.08-.70)	41.3 (3.6)	40.3 (3.1)	.25 (−.05-.54)
RPE (6–20)	11.6 (2.2)	11.6 (2.3)	−.01 (−.31-.29)	10.5^aa^ (2.2)	10.6^bb^ (2.1)	−.06 (−.39-.26)	11.5 (2.2)	11.4 (2.4)	.03 (−.33-.39)	10.5^a^ (2.1)	10.5 (2.0)	−.02 (−.38-.34)

Time to level time to peak exertion, *RR* respiratory rate, $\dot{\text{V}}$_T_ tidal volume, $\dot{\text{V}}$e/$\dot{\text{V}}$O_2_ ventilatory equivalent of oxygen, $\dot{\text{V}}$e/$\dot{\text{V}}$CO_2_ ventilatory equivalent of carbon dioxide, *HR* heart rate, *SBP* systolic pressure, *DBP* diastolic pressure, PP pulse pressure, RPP rate pressure product, *PETO*_*2*_ pressure end tidal O_2_, *PETCO*_*2*_ pressure end tidal CO_2_, *RPE* rating of perceived exertion (6–20)

*p ≤ 0.05, **p ≤ 0.01 between CPETs, ^a^*p* ≤ 0.05, ^aa^*p* ≤ 0.01 between groups for CPET-1, ^b^*p* ≤ 0.05, ^bb^*p* ≤ 0.01 between groups for CPET-2

Several measures at VAT during CPET-1 for the total sample were higher in CTL compared to ME/CFS including Work, TTL, $\dot{\text{V}}$O_2 (ml.kg-1.min-1)_, $\dot{\text{V}}$O_2 (ml/min)_, $\dot{\text{V}}$CO_2_, %peak $\dot{\text{V}}$O_2_, O_2_pulse (*p* ≤ 0.01), RER, HR, and lower RPE (*p* ≤ 0.05). Again, despite a low-active status of CTL, the differences were likely associated with the 15% higher $\dot{\text{V}}$O_2 (ml.kg-1.min-1)_ at VAT in CTL compared to ME/CFS. Differences between phenotypes for CPET-2 were also evident but occurred primarily due to a decline in measures in ME/CFS rather than an increase in CTL measures. Persistent higher measures in CTL remained for Work, TTL, $\dot{\text{V}}$O_2 (ml.kg-1.min-1)_, $\dot{\text{V}}$O_2 (ml/min)_, $\dot{\text{V}}$CO_2_, %peak $\dot{\text{V}}$O_2_, O_2_pulse, (*p* ≤ 0.01), and HR (*p* ≤ 0.05).

For matched-pairs, significant differences between phenotypes for CPET-1 did not differ from those in the total sample. As with the total sample, differences between phenotypes for CPET-2 occurred primarily due to declines in CPET-2 measures in ME/CFS and were largely consistent with phenotype differences for CPET-1. Although pairs were matched for peak $\dot{\text{V}}$O_2_, $\dot{\text{V}}$O_2_@VAT was 15% higher in CTL for CPET-1 (*p* ≤ 0.01) and 20% higher for CPET-2 (*p* ≤ 0.01). This was due to a 6.7% decline in $\dot{\text{V}}$O_2_@VAT in ME/CFS from CPET-1 to CPET-2 (*p* ≤ 0.05), with no change for CTL. These results indicate that $\dot{\text{V}}$O_2_@VAT represents a pivotal shift in O_2_ transport, and possibly energy metabolism, that may confer a greater negative impact on peripheral oxygen extraction (O_2_pulse) with a higher perception of effort (RPE) in ME/CFS compared to CTL.

### Derived measures of cardiac and pulmonary performance

Measures of cardiac and pulmonary performance derived from CPET variables for the total and the matched-pairs sample appear in Table 4. These include indices of maximum effort, measures at VAT, and gas exchange efficiency.

**Table 4:** Cardiac and pulmonary performance variables derived from CPET measures at peak effort and VAT for ME/CFS and Controls for CPET-1 and CPET-2

	Total Sample						VO_2_peak-matched pairs					
	ME/CFS (n = 84)			Controls (n = 71)			ME/CFS (n = 55)			Controls (n = 55)		
	CPET-1	CPET-2	ES (CI)	CPET-1	CPET-2	ES (CI)	CPET-1	CPET-2	ES (CI)	CPET-1	CPET-2	ES (CI)
%predicted HR_max_	90.0 (0.1)	87.7 (0.1)	.24^*^ (.03-.45)	90.4 (8.5)	89.8 (7.4)	.07 (−.15-.28)	90.1 (10.4)	87.2 (10.5)	.32^**^ (.07-.56)	91.4 (7.8)	90.4 (7.2)	.11 (−.13-.34)
%HRR_adjusted_	80.6 (19.3)	76.1 (21.1)	.25^**^ (.05-.44)	82.2 (16.4)	80.9 (13.9)	.07 (−.14-.28)	81.1 (20.2)	75.9 (20.6)	.26^*^ (.03-.49)	80.6 (20.1)	75.6 (20.6)	.25^*^ (.02-.48)
CTI_peak_	.90 (.10)	.88 (.11)	.21^*^ (.01-.41)	.90 (.08)	.89 (.08)	.10 (−.12-.32)	.90 (.11)	.87 (.11)	.32^**^ (.08-.56)	.91 (.08)	.90 (.07)	.12 (−.11-.35)
CTI_VAT_	.91 (.09)	.90 (.09)	.05 (−.26-.35)	.90 (.07)	.91 (.07)	−.08 (−.42-.25)	.91 (.10)	.90 (.09)	.01 (−.34-.36)	.91 (.07)	.91 (.07)	.02 (−.34-.37)
%predVO_2peak_	80.2 (18.1)	75.7 (19.2)	.25^**^ (.09-.41)	84.7 (18.3)	83.1 (17.9)	.09 (−.08-.25)	82.4 (15.4)	77.9 (17.9)	.26^**^ (.07-.45)	84.9 (18.7)	83.5^bb^ (17.6)	.08 (−.10-.26)
%VO_2peak_@VAT	50.0 (9.1)	50.0 (9.7)	.02 (−.24-.28)	54.2^a^ (8.3)	53.8^b^ (8.4)	.05 (−.23-.33)	48.3 (8.6)	48.5 (10.0)	−.02 (−.34-.31)	55.7^aa^ (8.3)	54.7^bb^ (8.4)	.11 (−.22-.44)
%HR_peak_ @VAT	68.4 (10.3)	69.5 (9.5)	−.12 (−.36-.12)	70.9 (8.4)	71.0 (7.3)	−.01 (−.27-.25)	67.7 (10.8)	69.2 (10.2)	−.16 (−.46-.14)	71.6 (8.2)	71.5 (7.3)	.01 (−.29-.31)
%Work_peak_@VAT	41.0 (14.3)	39.0 (14.9)	.14 (−.14-.43)	47.4^a^ (13.6)	46.7^bb^ (12.7)	.05 (−.26-.36)	40.3 (14.8)	39.7 (15.5)	.05 (−.32-.41)	48.9^a^ (14.5)	47.7^b^ (12.1)	.08 (−.28-.45)
%predVO_2peak_@VAT	40.1 (12.0)	37.4 (11.2)	.23^**^ (.07-.39)	45.8^aa^ (12.0)	44.6^bb^ (12.1)	.10 (−.07-.27)	39.9 (10.8)	37.6 (11.2)	.20^**^ (.02-.39)	47.2^aa^ (12.5)	45.8^bb^ (12.6)	.12 (−.06-.31)
$\dot{\text{V}}$ e/$\dot{\text{V}}$ CO_2VAT_	28.6 (3.4)	29.6 (4.0)	−.33^**^ (−.52-−.13)	28.8 (2.7)	29.5 (2.9)	−.22^*^ (−.43-−.01)	29.4 (3.3)	30.6 (4.0)	−.39^**^ (−.64-.14)	27.6^a^ (2.8)	28.3^bb^ (2.9)	−.22^*^ (−.46-.02)
OUES	1477.0 (409.5)	1417.2 (399.2)	.14^*^ (−.02-.31)	1712.4^aa^ (416.5)	1677.4^bb^ (442.6)	.08 (−.09-.26)	1516.5 (404.0)	1463.1 (340.0)	.13 (−.08-.33)	1732.9^aa^ (436.4)	1710.1^bb^ (460.4)	.05 (−.15-.25)
OUES_BSA_	788.7 (177.4)	760.2 (191.1)	.16 (−.03-.35)	887.5^aa^ (166.7)	867.9^bb^ (182.6)	.11 (−.10-.32)	809.6 (166.1)	785.7 (185.1)	.14 (−.10-.37)	875.5^a^ (162.0)	864.2^b^ (182.8)	.07 (−.17-.30)
%predicted OUES	68.5 (14.0)	66.0 (16.1)	.16 (−.03-.35)	74.7 (15.1)	73.0^b^ (16.4)	.11 (−.10-.31)	70.0 (13.7)	68.1 (16.7)	.12 (−.11-.35)	76.2^a^ (15.3)	75.0^bb^ (16.7)	.07 (−.15-.30)

*%pred HR*_*max*_ actual peak HR/(220-age); *%HRR*_*adjusted*_ HRR/(predicted HR_max_-HR_rest_), *CTI* chronotropic index, *%predVO*_*2peak*_ actual VO_2peak_/predicted VO_2peak_, *%predVO*_*2peak*_*@VAT* actual VO_2_@VAT/predicted VO_2peak_; $\dot{V}$* e/* $\dot{V}$* CO*_*2VAT*_ ventilatory equivalent of carbon dioxide at VAT, *OUES* oxygen uptake efficiency slope, *OUES*_*BSA*_ OUES adjusted for body surface area; %predicted OUES actual OUES/predicted OUES

*p ≤ 0.05, **p ≤ 0.01 between CPETs, ^a^*p* ≤ 0.05, ^aa^*p* ≤ 0.01 between groups for CPET-1, ^b^*p* ≤ 0.05, ^bb^*p* ≤ 0.01 between groups for CPET-2.

#### Maximum effort

One indicator of maximum effort during incremental exercise, %predicted HR_max_, did not differ between phenotypes for the total sample or matched-pairs, with both groups reaching the required threshold of ≥ 85% of age-predicted HR_max_. However, compared to CPET-1, %predicted HR_max_ for ME/CFS was significantly lower during CPET-2 in both the total sample (*p* ≤ 0.05) and matched-pairs (*p* ≤ 0.01), but still ≥ 85% of age-predicted HR_max_. Another indicator of maximum exercise effort and cardiac performance, age-adjusted percent heart rate reserve (%HRR_adjusted_), unlike %predicted HR_max_, accounts for an individual’s range of HR responses from resting to maximum HR. Similarly, there were no differences between phenotypes for the total sample or matched-pairs, with both phenotypes achieving the threshold level of ≥ 80% of HRR_adjusted_ during CPET-1. However, for CPET-2, there was a significant decline in %HRR_adjusted_ for ME/CFS in the total sample (*p* ≤ 0.01) and matched-pairs (*p* ≤ 0.05) that fell below the threshold of 80%, suggesting that maximum effort was not given during CPET-2. Interestingly, a similar decline in %HRR_adjusted_ during CPET-2 was observed in the matched-pairs CTL as well (*p* ≤ 0.05).

Chronotropic incompetence (CI) is the inability to increase heart rate commensurate with metabolic demand and can contribute to exertion intolerance [52]. Several formulas are proposed to assess chronotropic index (CTI) although the formula used here to calculate CTI at VAT and peak exertion may best describe the relationship between heart rate and metabolic demand [53]:

$${\text{Estimated}} {\text{HR}}_{{{\text{stage}}}} \, = \,[({22}0\, - \,{\text{age}}\, - \,{\text{HR}}_{{{\text{rest}}}} )]\, \times \,[({\text{METs}}_{{{\text{stage}}}} \, - \,{1})/({\text{METs}}_{{{\text{peak}}}} \, - \,{1})]\, + \,{\text{HR}}_{{{\text{rest}}}}$$

CTI_peak_ and CTI_VAT_ were calculated using the MET values at the stages of peak $\dot{\text{V}}$O_2_ and $\dot{\text{V}}$O_2_@VAT, respectively. As with %predicted HR_max_ and %HRR_adjusted_, CTI_peak_ did not differ between phenotypes for either CPET, but did decrease in ME/CFS on CPET-2 for the total (*p* ≤ 0.05) and matched-pairs (*p* ≤ 0.01) samples.

Percent predicted $\dot{\text{V}}$O_2_peak (%pred $\dot{\text{V}}$O_2peak_) was calculated as $\dot{\text{V}}$O_2_peak measured from CPET-1 divided by $\dot{\text{V}}$O_2_peak predicted based on age and sex [54]. It decreased in the total group of ME/CFS from 80.2% to 75.7% during CPET-2 (*p* ≤ 0.01) but not in CTL. Similar results were evident in the matched-pairs sample with a significant decline in %pred $\dot{\text{V}}$O_2peak_ for ME/CFS on CPET-2 (*p* ≤ 0.01), but not in CTL. Both groups achieved values higher than typical of heart failure and cardiac patients at increased risk of death (< 50% pred $\dot{\text{V}}$O_2peak_) [55, 56]. However, due to a decline during CPET-2 to 77.9% in matched-pairs ME/CFS , %pred $\dot{\text{V}}$O_2peak_ for matched-pairs CTL (83.5%) was higher (*p* ≤ 0.01).

#### Measures at VAT

Measures at VAT, the point during incremental exercise when energy production begins to rely increasingly on anaerobic metabolism to meet energy demands, are particularly relevant for those with ME/CFS. This transition of metabolic energy production is often associated with symptom exacerbation and perceived exertion in ME/CFS.

Unlike CTI_peak_, CTI_VAT_ did not differ between phenotypes or CPETs for either the total or matched-pairs groups. Thus, the apparent mismatch in cardiac response to metabolic demand observed at peak effort is not apparent at VAT but begins to deteriorate in ME/CFS at metabolic demands above VAT.

The percentage of peak $\dot{\text{V}}$O_2_ at which VAT occurs (%$\dot{\text{V}}$O_2peak_@VAT) is typically between 45–65% [57], although may be higher in athletes or lower in cardiac, pulmonary and other diseases [58]. There was no difference in %$\dot{\text{V}}$O_2peak_@VAT between CPETs for either phenotype but was consistently lower in ME/CFS compared to CTL on both CPETs for the total (*p* ≤ 0.05) and matched-pair samples (*p* ≤ 0.01). However, $\dot{\text{V}}$O_2_@VAT expressed as a percentage of predicted $\dot{\text{V}}$O_2peak_ (%_pred_ $\dot{\text{V}}$O_2peak_@VAT) revealed very low VAT threshold in ME/CFS. With values at or under 40% in both the total and matched-pair samples, ME/CFS was well below normal range values of CTL. Because HR generally tracks linearly with VO_2_ during incremental exercise until near peak exertion, it was anticipated that the percentage of peak HR at VAT (%HR_peak_@VAT) would respond similarly to %$\dot{\text{V}}$O_2peak_@VAT. In fact, there were no significant differences between phenotypes, CPETs, or sample groups. There was, however, an overall non-significant trend of lower %HR_peak_@VAT in ME/CFS compared to CTL. In contrast, the percentage of peak Work at which VAT occurred (%Work_peak_@VAT) was 14% and 16% lower in the total sample of ME/CFS compared to CTL for CPET-1 (*p* ≤ 0.05) and CPET-2 (*p* ≤ 0.01), respectively, and 16% lower in the matched-pairs for both CPETs (*p* ≤ 0.05). Thus, the magnitude of work performed at the VAT level of oxygen consumption declined notably in ME/CFS during CPET-2.

#### Gas exchange efficiency

The ability to ventilate sufficiently to eliminate CO_2_ can be described by the ratio of minute ventilation and CO_2_ production, or $\dot{\text{V}}$e/$\dot{\text{V}}$CO_2_. If expressed as a ratio, it is least variable when measured at a time between the VAT and point of ventilatory compensation (VCP). When expressed as a slope value, it is determined from the start of incremental exercise to the VCP. Whether ratio or slope, a value higher than age/sex normal values indicates impaired ventilatory efficiency. Both the ratio and slope are highly similar, although the ratio tends to be less variable [58]. Measured at VAT, the $\dot{\text{V}}$e/$\dot{\text{V}}$CO_2VAT_ ratio increased during CPET-2 similarly for ME/CFS (*p* ≤ 0.01) and CTL (*p* ≤ 0.05) in the total sample. Likewise, $\dot{\text{V}}$e/$\dot{\text{V}}$CO_2VAT_ in the matched-pairs increased during CPET-2 for ME/CFS for (*p* ≤ 0.01) and CTL (*p* ≤ 0.05). However, only in the matched-pairs was $\dot{\text{V}}$e/$\dot{\text{V}}$CO_2VAT_ significantly higher in ME/CFS compared to CTL for CPET-1 (*p* ≤ 0.05) and CPET-2 (*p* ≤ 0.01).

Oxygen Uptake Efficiency Slope (OUES) is derived from the relationship between $\dot{\text{V}}$O_2_ and log-transformed $\dot{\text{V}}$e, such that [59]:

$$\mathop V\limits^{.}O_{2} \, = \,a \, log \mathop V\limits^{.}e\, + \,b, \, where \, a\, = \,OUES$$

OUES is an index of cardiopulmonary functional reserve or the ventilatory requirement for a given $\dot{\text{V}}$O_2_. The OUES is independent of exercise intensity so it is not necessary that maximal effort is achieved during an incremental exercise test, only that several submaximal exercise workloads are completed. For that reason, OUES is a valuable index to measure, especially when testing those who are unable or for whom it is of high risk to work to maximum effort, such as orthopedic limitations, disease severity or morbid obesity. However, OUES is a relevant indicator of cardiopulmonary function in a broader population as well [60].

OUES was 13% to 16% lower in ME/CFS compared to CTL in both sample groups for both CPETs (*p* ≤ 0.01). Only in the total sample was OUES lower on CPET-2 compared to CPET-1 (*p* ≤ 0.05). When corrected for body surface area (OUES_BSA_), significant differences persisted, albeit smaller, between phenotypes for CPET-1 with 11% (CPET-1; *p* ≤ 0.01) and 13% (CPET-2; *p* ≤ 0.01) lower OUES_BSA_ for ME/CFS compared to CTL. Similarly in the matched-pairs, OUES_BSA_ was 8% (CPET-1; *p* ≤ 0.05) and 11% (CPET-2; *p* ≤ 0.01) lower in ME/CFS. OUES expressed relative to OUES predicted for age/sex (based on predicted $\dot{\text{V}}$O_2_ and predicted $\dot{\text{V}}$e), %predicted OUES, was significantly lower during CPET-2 for the total ME/CFS sample (*p* ≤ 0.05). For the matched-pairs sample, %predicted OUES was lower in ME/CFS for CPET-1 (*p* ≤ 0.05) and CPET-2 (*p* ≤ 0.01). Collectively, all expressions of OUES indicate that the cardiopulmonary functional reserve in ME/CFS is compromised compared to sedentary controls even in ME/CFS matched with CTL for aerobic capacity.

### Intraclass correlation coefficients

Intraclass correlation coefficients (ICC) were calculated to assess reliability of 20 CPET variables at peak exercise for ME/CFS and CTL in the total and matched-pair samples (Table 5). ICCs with moderate^*^ reliability across tests (0.50 – 0.75), good^**^ reliability (0.75 – 0.90), and excellent^***^ reliability (> 0.90) [61, 62] are denoted. At first glance, it is apparent that ICCs with *excellent* reliability occur more frequently in the total sample of CTL comprising 20% of all variables, compared to none in ME/CFS. Despite all participants giving maximum effort during both tests, the ICCs for ME/CFS are comparatively lower ranging from *good* to *moderate*. Similarly for the matched-pairs, ICCs for CTL included 20% with *excellent* reliability and only one variable (O_2_pulse) in the *excellent* range for ME/CFS. Considering both *good* and *excellent* ICCs collectively, 75% (total sample) and 80% (matched pairs) of the variables reported in this table reached 0.75 or higher and demonstrate the overall normally high stability of peak CPET measures in those who do not have ME/CFS.

**Table 5:** Intraclass correlation coefficients (ICC) between CPET-1 and CPET-2 at peak VO_2_ for ME/CFS and Controls

	Total sample				VO_2_peak-matched pairs			
	ME/CFS (n = 84)		Controls (n = 71)		ME/CFS (n = 55)		Controls (n = 55)	
Variable at Peak	ICC	95% CI	ICC	95% CI	ICC	95% IC	ICC	95% CI
Work (W)	0.87^**^	.78–.92	0.95^***^	.92–.97	0.80^**^	.67–.87	0.95^***^	.92–.97
Time to level (sec)	0.83^**^	.72–.89	0.87^**^	.80–.92	0.73^*^	.56–.83	0.86^**^	.78–.92
RER	0.65^*^	.50–.76	0.60^*^	.42–.73	0.63^*^	.50–.73	0.65^*^	.47–.78
$\dot{\text{V}}$e (L/min)	0.82^**^	.70–.89	0.87^**^	.80–.92	0.81^**^	.67–.88	0.88^**^	.81–.93
RR (breaths/min)	0.74^*^	.63–.82	0.76^**^	.65–.85	0.74^*^	.65–.82	0.78^**^	.65–.86
$\dot{\text{V}}$_T_ (L/min)	0.85^**^	.76–.90	0.90^***^	.84–.93	0.84^**^	.77–.90	0.93^***^	.88–.96
$\dot{\text{V}}$O_2_ (ml^.^kg^−1.^min^−1^)	0.86^**^	.75–.92	0.88^**^	.82–.92	0.82^**^	.65–.90	0.91^***^	.84–.94
$\dot{\text{V}}$O_2_ (ml/min)	0.87^**^	.77–.92	0.91^***^	.85–.94	0.86^**^	.69–.92	0.93^***^	.88–.96
$\dot{\text{V}}$CO_2_ (ml/min)	0.84^**^	.69–.91	0.88^**^	.81–.92	0.78^**^	.59–.87	0.90^**^	.82–.94
$\dot{\text{V}}$e/$\dot{\text{V}}$O_2_	0.71^*^	.58–.80	0.68^*^	.53–.79	0.70^*^	.59–.78	0.70^*^	.54–.82
$\dot{\text{V}}$e/$\dot{\text{V}}$ CO_2_	0.74^*^	.63–.83	0.79^**^	.69–.86	0.77^**^	.68–.84	0.79^**^	.67–.87
HR (bpm)	0.79^**^	.67–.86	0.79^**^	.69–.86	0.80^**^	.65–.87	0.85^**^	.75–.91
O_2_pulse ($\dot{\text{V}}$O_2_/HR)	0.87^**^	.81–.92	0.90^***^	.85–.94	0.88^***^	.81–.93	0.92^***^	.87–.95
SBP (mmHg)	0.86^**^	.79–.91	0.85^**^	.77–.90	0.84^**^	.78–.89	0.87^**^	.79–.92
DBP (mmHg)	0.71^*^	.58–.81	0.73^*^	.60–.82	0.73^*^	.62–.81	0.79^**^	.66–.87
PP (mmHg)	0.76^**^	.65–.84	0.78^**^	.67–.86	0.72^**^	.61–.80	0.79^**^	.66–.87
RPP	0.83^**^	.74–.89	0.80^**^	.70–.87	0.82^**^	.71–.89	0.85^**^	.75–.91
PETO_2_ (mmHg)	0.75^**^	.64–.83	0.70^*^	.55–.80	0.77^**^	.68–.84	0.67^*^	.49–.79
PETCO_2_ (mmHg)	0.80^**^	.71–.87	0.78^**^	.66–.85	0.82^**^	.75–.87	0.78^**^	.65–.87
RPE (6–20)	0.54^*^	.37–.67	0.59^*^	.41–72	0.50^*^	.35–.63	0.56^*^	.35–.72

Time to level = time to peak exertion, *RER* respiratory exchange ratio, $\dot{\text{V}}$e minute ventilation, *RR* respiratory rate, $\dot{\text{V}}$_T_ tidal volume. $\dot{\text{V}}$e/$\dot{\text{V}}$O_2_ ventilatory equivalent of oxygen, $\dot{\text{V}}$e/$\dot{\text{V}}$CO_2_ ratio of ventilatory equivalent of carbon dioxide, *HR* heart rate, *SBP* systolic pressure, *DBP* diastolic pressure, *PP*  pulse pressure, *RPP* rate pressure product, *PETO*_*2*_ pressure end tidal O_2_, *PETCO*_*2*_ pressure end tidal CO_2_, *RPE* rating of perceived exertion (6–20)

*moderate reliability, **good reliability, ***excellent reliability

Of particular interest are measures at VAT (Table 6) with overall lower reliability in the total sample of ME/CFS compared to CTL for gas exchange measures ($\dot{\text{V}}$e, $\dot{\text{V}}$O_2_, $\dot{\text{V}}$CO_2_) compared to equal or higher ICCs in ME/CFS for hemodynamic measures (SBP, DBP, PP). Overall, *good* to *excellent* reliability was evident in only 20–25% of the 20 measured variables for ME/CFS in the total and matched pairs samples in contrast to 55% (11/20) of variables for CTL in both sample groups. The marked differences in ICCs between phenotypes further substantiate the challenge of those with ME/CFS to reproduce CPET measures that are known to be highly reproducible in healthy [63], athletic [64], and diseased [65, 66] populations.

**Table 6:** Intraclass correlation coefficients (ICC) between CPET-1 and CPET-2 at ventilatory/anaerobic threshold (VAT) for ME/CFS and Controls

Variable at VAT	Total sample				VO_2_peak-matched pairs			
	ME/CFS (n = 84)		Controls (n = 71)		ME/CFS (n = 55)		Controls (n = 55)	
	ICC	95% CI	ICC	95% CI	ICC	95% CI	ICC	95% CI
Work (W)	0.64^*^	.49–.75	0.76^**^	.64–.84	0.50^*^	.27–.67	0.81^**^	.69–.88
Time to level (sec)	0.62^*^	.47–.73	0.76^**^	.65–.85	0.48	.25–.66	0.82^**^	.70–.89
RER	0.67^*^	.53–.77	0.46	.26–.63	0.62^*^	.43–.76	0.45	.21–.64
$\dot{\text{V}}$e (L/min)	0.65^*^	.51–.76	0.83^**^	.75–.89	0.65^*^	.47–.78	0.87^**^	.78–.92
RR (breaths/min)	0.58^*^	.42–.71	0.58^*^	.40–.71	0.59^*^	.39–.74	0.59^*^	.39–.74
$\dot{\text{V}}$_T_ (L/min)	0.70^*^	.58–.80	0.80^**^	.70–.87	0.72^*^	.56–.82	0.79^**^	.67–.87
$\dot{\text{V}}$O_2_ (ml^.^kg^−1.^min^−1^)	0.72^*^	.58–.81	0.90^***^	.84–.93	0.68^*^	.49–.80	0.92^***^	.85–.95
$\dot{\text{V}}$O_2_ (ml/min)	0.76^**^	.66–.85	0.92^***^	.87–.95	0.77^**^	.62–.86	0.92^***^	.87–.95
$\dot{\text{V}}$CO_2_ (ml/min)	0.69^*^	.55–.79	0.84^**^	.76–.90	0.64^*^	.45–.77	0.85^**^	.75–.91
$\dot{\text{V}}$ e/$\dot{\text{V}}$O_2_	0.68^*^	.55–.78	0.60^*^	.43–.73	0.70^*^	.53–.81	0.63^*^	.44–.77
$\dot{\text{V}}$e/$\dot{\text{V}}$CO_2_	0.73^*^	.52–.84	0.75^**^	.63–.84	0.71^*^	.48–.84	0.80^**^	.63–.89
HR (bpm)	0.60^*^	.45–.72	0.67^*^	.52–.78	0.60^*^	.40–.75	0.69^*^	.52–.81
O_2_pulse ($\dot{\text{V}}$O_2_/HR)	0.90^**^	.86–.92	0.93^***^	.89–.96	0.89^***^	.79–.94	0.95^***^	.91–.97
SBP (mmHg)	0.80^**^	.69–.87	0.68^*^	.53–.79	0.82^**^	.70–.89	0.67^*^	.50–.79
DBP (mmHg)	0.76^**^	.63–.84	0.80^**^	.70–.87	0.74^*^	.59–.84	0.82^**^	.67–.89
PP (mmHg)	0.72^*^	.59–.81	0.55^*^	.36–.69	0.64^*^	.46–.77	0.52^*^	.30–.69
RPP	0.68^*^	.54–.78	0.56^*^	.37–.70	0.64^*^	.46–.77	0.58^*^	.38–.73
PETO_2_ (mmHg)	0.68^*^	.55–.78	0.66^*^	.51–.78	0.72^*^	.56–.82	0.66^*^	.48–.78
PETCO_2_ (mmHg)	0.76^**^	.57–.86	0.80^**^	.70–.87	0.75^**^	.49–.87	0.82^**^	.63–.90
RPE (6–20)	0.64^*^	.50–.75	0.65^*^	.50–.77	0.66^*^	.49–.79	0.71^*^	.55–.82

Time to level = time to peak exertion; *RER* respiratory exchange ratio, $\dot{\text{V}}$e   minute ventilation, *RR* respiratory rate, $\dot{\text{V}}$_T_ tidal volume, $\dot{\text{V}}$e/$\dot{\text{V}}$O_2_  ventilatory equivalent of oxygen, $\dot{\text{V}}$e/$\dot{\text{V}}$CO_2_   ratio of ventilatory equivalent of carbon dioxide, *HR* heart rate; *SBP*  systolic pressure, *DBP* diastolic pressure, *PP*  pulse pressure, *RPP*  rate pressure product, *PETO*_*2*_ pressure end tidal O_2_, *PETCO*_*2*_ pressure end tidal CO_2_, *RPE* rating of perceived exertion (6–20); *moderate reliability, **good reliability, ***excellent reliability.

### Impairment status

**Fig. 5:**
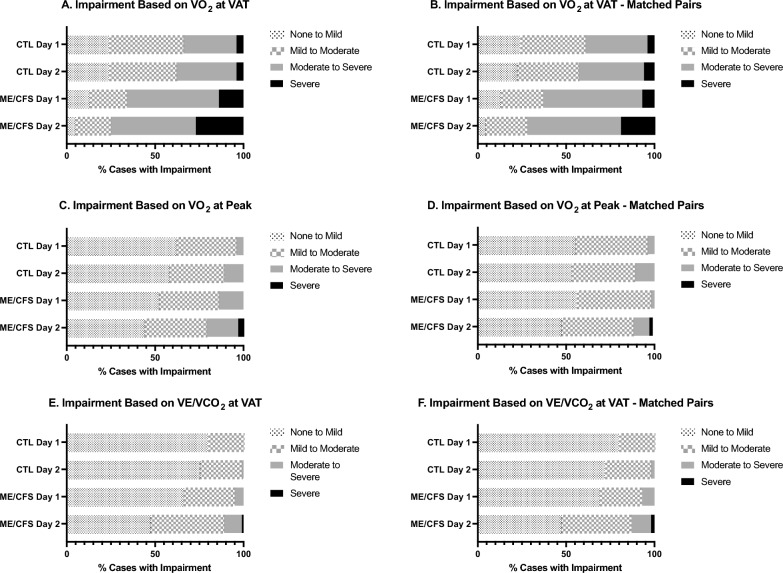
Clinical impairment of ME/CFS and controls during CPET-1 and CPET-2. Impairment status of ME/CFS (n = 84) and controls (CTL; n = 71), and VO_2_peak-matched pairs (n = 55) was assigned using established impairment ratings of Weber & Janicki (1985) and Arena & Sietsema (2011) for VO_2_ at ventilatory/anaerobic threshold (VAT; **A**,** B**), VO_2_peak (**C**,** D**) and VE/VCO_2_ at VAT (**E**,** F**). Data are expressed as percentage of cases within each of the impairment classifications for CPET-1 and CPET-2

Clinical impairment status was determined using established impairment ratings for $\dot{\text{V}}$O_2_peak, $\dot{\text{V}}$O_2_@VAT [67] and $\dot{\text{V}}$e/$\dot{\text{V}}$CO_2_@VAT [68] for the total sample and matched-pairs for CPET-1 and CPET-2 (Fig. 5A–F). Impairment categories ranged from *none to mild*, *mild to moderate*, *moderate to severe*, and *severe*.

Figures 5A–C illustrate the similarity between phenotypes in impairment status at baseline (CPET-1) for all three indices of impairment. Based on $\dot{\text{V}}$O_2_peak, 62% of CTL and 52% of ME/CFS had *none to mild* impairment, suggesting that the CTL in this study were indeed, sedentary and low active, and similar in functional capacity to ME/CFS at baseline. While no participants for either phenotype met the *severe* impairment category, 14% of ME/CFS met *moderate to severe* impairment compared to only 4% of CTL. Based on $\dot{\text{V}}$O_2_@VAT, phenotype differences were more striking. Most CTL (42%) identified as *mild to moderate* impairment whereas the majority of ME/CFS (52%) were in the *moderate to severe* category. Additionally, 14% of ME/CFS classified as *severe* impairment compared to only 4% of CTL. Lastly, using $\dot{\text{V}}$e/$\dot{\text{V}}$CO_2_@VAT impairment standards, 80% of CTL and 66% of ME/CFS fell into *none to mild* impairment. The remaining 20% of CTL compared to 29% of ME/CFS showed *mild to moderate* impairment with 5% of ME/CFS rated as *moderate to severe*. Overall, baseline data from CPET-1 suggests that ME/CFS and CTL were comparable regarding impairment status based on $\dot{\text{V}}$O_2_peak and $\dot{\text{V}}$e/$\dot{\text{V}}$CO_2_@VAT, but less so for $\dot{\text{V}}$O_2_@VAT where a greater severity of impairment in ME/CFS was apparent.

Post-exertional data (CPET-2) reveals a shift toward increasingly severe impairment in ME/CFS compared to CTL for all three indices. Using $\dot{\text{V}}$O_2_peak, 56% of ME/CFS were impaired ranging in severity from *mild to severe* (including *mild to moderate*, *moderate to severe*, and *severe*) compared to 48% from CPET-1. In contrast, there was little appreciable shift in impairment status in CTL from CPET-1 to CPET-2. Notably, marked shifts in impairment status of ME/CFS were most evident based on $\dot{\text{V}}$O_2_@VAT, with 95% of cases ranging from *mild to severe* compared to 87% based on CPET-1. Importantly, the percentage of ME/CFS rated as *severe* from CPET-2 (27%) was doubled compared to *severe* cases based on CPET-1 (14%), illustrating the deleterious impact on the gas exchange threshold (VAT) due to exertion intolerance in ME/CFS. In contrast, only a slight increase was observed in CTL in *moderate to severe* plus *severe* impairment cases from CPET-1 (34%) to CPET-2 (38%). Finally, impairment based on $\dot{\text{V}}$e/$\dot{\text{V}}$CO_2_@VAT revealed a similar trend of worsening impairment status for ME/CFS with an overall increase in *mild to severe* cases on CPET-2 and only a slight increase in impairment severity for CTL.

Figures 5D–F for the matched-pairs show impairment ratings based on $\dot{\text{V}}$O_2_peak, $\dot{\text{V}}$O_2_@VAT and $\dot{\text{V}}$e/$\dot{\text{V}}$CO_2_@VAT. Similar trends discussed above for the total sample were evident for ME/CFS compared to CTL from CPET-1 to CPET-2. Because pairs were matched on $\dot{\text{V}}$O_2_peak, not surprisingly, impairment status for both phenotypes based on CPET-1 were remarkably similar. Still, however, impairment ratings based on CPET-2 reveal an increase in impairment severity of ME/CFS ranging from *mild to severe* (including *mild to moderate*, *moderate to severe*, and *severe*) compared to little change in CTL. Shifts in impairment ratings based on $\dot{\text{V}}$O_2_@VAT and $\dot{\text{V}}$e/$\dot{\text{V}}$CO_2_@VAT from CPET-2 in the matched-pairs were consistent with those discussed above for the total sample suggesting that matching participants based on $\dot{\text{V}}$O_2_peak had little impact on the post-exertional (CPET-2) decline in energy metabolism in ME/CFS.

## Discussion

The primary objectives of this largest study to date of a 2-day CPET protocol in ME/CFS were to, (1) characterize the baseline aerobic capacity of ME/CFS, (2) assess reproduction of CPET variables as an indicator of recovery following exertion, (3) characterize the magnitude of impairment in ME/CFS based on CPET measures, and (4) compare the cardiovascular, pulmonary, and musculoskeletal contributors to aerobic capacity, energy production, and recovery following exertion between ME/CFS and sedentary controls. Secondary objectives were to compare CPET variables in a subset of ME/CFS and sedentary controls matched for sex, age, and peak oxygen consumption, and to assess the consistency and reproducibility of cardiopulmonary and metabolic variables across two CPETs. The discussion is organized as follows: Peak exercise (CPET-1 total group, matched pairs; CPET-2 total group, matched pairs); Ventilatory/Anaerobic Threshold; Derived Measures; ICCs; Impairment Status.

### Peak exercise

For the total sample, CPET-1 (baseline) measures at peak exercise (Table 2) for ME/CFS were consistent with previous reports [69–72] that indicate an overall lower capacity to do work, shorter time to peak effort, reduced ventilatory function, O_2_ consumption, CO_2_ production, and oxygen pulse compared to controls. Recent work by Cook et al.[30] included 178 ME/CFS and 169 controls who completed a single CPET, the results of which were comparable to the total sample in the present study. However, when they matched 99 ME/CFS with controls for age and peak $\dot{\text{V}}$O_2_, some differences in the total sample disappeared, leaving only four measures related to ventilatory function (respiratory rate (RR), tidal volume (V_T_), and ventilatory equivalents of $\dot{\text{V}}$O_2_ and $\dot{\text{V}}$CO_2_) to distinguish phenotypes. In contrast, phenotype differences in the total sample of this study persisted in the analysis of the 55 matched-pairs sample. The discrepant findings in the matched pairs of Cook et al. and the present data may possibly be explained by differences in matching ME/CFS with CTL. While both studies matched pairs based on age and peak $\dot{\text{V}}$O_2_, 11 of the 99 pairs in the Cook et al. study included males matched with females. In general, sex differences in oxygen carrying capacity and blood flow, such as cardiac dimensions [48], vessel diameter[73], pulse oximetry [49], blood volume [74], 2,3 DPG [75], and hemoglobin [47] contribute to an overall greater oxygen carrying capacity in males [74]. Thus, matching males with females for VO_2_peak would not control for sex differences in measures that could directly impact oxygen consumption (e.g., blood volume, hemoglobin, 2,3 DPG, etc.).

One additional difference observed in ME/CFS of the matched-pairs sample was a lower pulse pressure, or the difference between SBP and DBP. Like the present study, Cook et al. reported an overall higher DBP in ME/CFS at rest in both the total and matched samples. While higher DBP could contribute to a reduced pulse pressure at rest, blood pressure during exercise was not reported so it is only speculative as to whether a lower pulse pressure remained during exercise. In the present study, neither SBP or DBP at peak exercise differed significantly between phenotypes, but SBP was lower and DBP was higher in ME/CFS compared to CTL. Collectively, these differences resulted in an overall significantly narrowed pulse pressure at peak effort in ME/CFS (p ≤ 0.01), increasing total peripheral resistance (TPR) and contributing to circulatory decompensation. Similar results of narrowed pulse pressure were observed in ME/CFS during a 10-min orthostatic stressor (NASA lean test) [76], and lower SBP with higher vagal tone were associated with higher risk of PEM [77]. Vascular dysregulation is increasingly attributed to hypoperfusion in ME/CFS [78]. As such, assessment of temporal shifts in hemodynamics during incremental exercise is critical when evaluating functional capacity in ME/CFS. Further, oxygen pulse is considered a non-invasive surrogate measure for stroke volume and relates to peripheral oxygen extraction. Reports of smaller cardiac size [79], preload failure [80], reduced peripheral oxygen delivery and endothelial dysfunction [81–83] support a reduction in energy metabolism in ME/CFS due to impaired blood flow and oxygen delivery.

A recent study of post-infectious ME/CFS (N = 17) and healthy controls (N = 21) assessed a comprehensive panel of physiological, physical, cognitive, biochemical, microbiological, and immunological variables [84]. Of these measures, only 8 ME/CFS and 9 controls completed a single CPET with an average VO_2_peak about 40% higher in the control group. Based on this small sample size and inappropriately matched control group, authors suggested that impaired ANS function in ME/CFS, evidenced by diminished HRV, abnormal tilt-related symptoms, and other abnormal orthostatic responses, led to lower metabolic energy production and work output, and may be contributed to by a reduced ‘effort preference’. Effort preference was assessed in this study using the Effort-Expenditure for Rewards Task [85], which utilized a small motor task to assess for anhedonia typically associated with major depressive disorder. The Effort-Expenditure for Rewards Task is not highly associated with measures of whole-body oxygen consumption or power output compared to conventional indices of effort (%peak HR, RER, RPE), none of which were reported in the study. Whereas the link between ANS dysfunction and impaired energy metabolism is not inconsistent with the systemic CPET data reported herein, their reasoning is misguided. It has long been known that the magnitude of cardiovascular responses to exertion is predominantly influenced by the relative level of muscle activation (number and intensity of activated muscle fibers) via feedback loop from peripheral interoceptors (e.g., Golgi tendon organs, muscle spindles, etc.) to the motor cortex then to the brainstem [85]. Disruption of this feedback loop at any level, for example, due to infection of the vagus nerve postulated by VanElzakker [86] to emanate from the gut of ME/CFS, would negatively impact this tightly controlled process and downregulate central nervous system signaling of cardiovascular support peripherally for energy production. Consequently, during incremental exercise (i.e., CPET) accumulation of local muscle metabolites from insufficient blood flow coupled with dysregulated central signaling at the brainstem, will directly inhibit the relative level of muscle activation and thereby reduce effort. Given that both ME/CFS and well-matched CTL in the present study achieved similarly high metrics at peak effort during CPET, we saw no evidence of reduced peak effort in ME/CFS. This, however, was not the case during submaximal exercise, discussed subsequently, with ME/CFS reporting a higher RPE at VAT compared to CTL for both CPETs. These findings illustrate the significance of the critical metabolic shift in energy metabolism that begins at VAT and the deleterious impact on perception of effort in ME/CFS during low and moderate intensity work that are consistent with activities of daily living.

Based on the present findings at peak exercise, lower oxygen pulse and narrowed pulse pressure suggests that impaired oxygen delivery during exercise distinguishes ME/CFS from CTL, even when matched for $\dot{\text{V}}$O_2_peak. Reports of reduced stroke volume in ME/CFS were attributed to reduced blood volume and possibly to smaller end-diastolic ventricular wall mass [87]. Low blood volume in ME/CFS has been described previously [88, 89] and, together with findings of endothelial dysfunction could impair peripheral feedback and disrupt brainstem signaling [90]. Autonomic tone was assessed in ME/CFS using heart rate variability (HRV) [91], heart rate recovery (HRR) [92], and other measures of heart rate during orthostatic stressors and following a 2-d CPET protocol. While not sufficient alone to be regarded as a biomarker of ME/CFS, heart rate indices signaled reduced vagal tone and increased sympathetic drive compared to controls. Dysregulated ANS function may well explain not only compromised blood flow and oxygen delivery, but also ventilatory dysfunction evidenced by low ventilatory measures in ME/CFS.

### Post-exertional measures at peak

The second test (CPET-2) assessed the capacity to reproduce CPET measures. Mentioned previously, the reproducibility of CPET measures is well-established [93–95] so CPET results are expected to be reproduced within normal variability with confirmation of maximum effort. Results from CPET-2 further substantiated the challenge of ME/CFS to recover normally following CPET-1. Despite meeting maximum effort criteria, the total sample of ME/CFS but not CTL, exhibited significant reductions in peak Work (− 5.5%), time to peak exercise (− 6.6%), ventilatory measures (− 4.9% to − 7.8%), heart rate (− 2.6%), O_2_ pulse (− 4.0%), and rate-pressure product (− 3.4%). In contrast for CTL, only $\dot{\text{V}}$CO_2_ declined significantly by 3% during CPET-2. Similarly, for ME/CFS in the matched-pairs, comparatively larger reductions in the same CPET measures were observed that ranged from − 3.2% to − 8.6%. There were no declines in matched CTL on CPET-2, indicating a normal recovery with no change in peak oxygen consumption or peak work. Other smaller studies using the 2-day CPET protocol reported similar findings of post-exertional reductions in peak measures of $\dot{\text{V}}$O_2_, work, ventilatory measures, and heart rate. Similarly, in studies with control subjects, CPET measures were reproduced normally [22, 28, 96].

### Ventilatory/Anaerobic threshold

Measures at VAT during CPET are highly relevant for athletes as well as for those with ME/CFS. Peak $\dot{\text{V}}$O_2_ is considered the best indicator of aerobic capacity [95, 97] or the maximum ability to produce energy to do work, but $\dot{\text{V}}$O_2_ at VAT is generally regarded as the best indicator of aerobic ‘performance’, or the intensity at which energy production can be sustained with limited fatigue-inducing consequences [98–100]. The intensity of work at which VAT occurs during incremental or graded exercise marks a shift in the balance of energy production toward increasing reliance on anaerobic processes to meet an increasing energy demand. Not without consequences, the faster rate of energy production afforded by anaerobic metabolism is accompanied by progressive accumulation of H^+^, lactate, CO_2_, a reduction of several glycolytic (anaerobic) rate-limiting enzymes (e.g., hexokinase, phosphofructokinase, etc.), along with other metabolic efforts to reduce NADH to NAD + for energy metabolism to continue. Energy production at intensities that are consistent with or exceed VAT will ultimately be limited by anaerobic metabolic by-products and constrain work output. For the athlete, knowing heart rate or performance velocity at VAT is valuable to gauge the ‘time’ spent exerting above VAT during a race so as not to suffer a decline in performance prior to crossing the finish line [101]. However, for ME/CFS, where the VAT threshold is markedly lower compared even to sedentary counterparts [23, 28], exceeding this point of metabolic shift occurs at a comparatively lower workload such that for many, even normal-speed walking will precipitate fatigue. Severely ill ME/CFS may exceed the VAT level of energy production simply rising from bed or brushing teeth. For many, activities of daily living (ADLs) may exceed VAT, aggravate fatigue, and exacerbate symptoms of PEM [19, 25].

As with CPET measures at peak exercise, baseline measures at VAT (Table 3) for the total sample of ME/CFS compared to CTL revealed a lower work output, shorter time to reach VAT, and lower O_2_ consumption, CO_2_ production, heart rate, and oxygen pulse. Likewise, Cook et al. [30] reported similar results for the total sample except for oxygen pulse (unchanged) and time to VAT (not reported). Although trending lower in ME/CFS, ventilatory measures at VAT ($\dot{\text{V}}$e, RR, $\dot{\text{V}}$t) were not significantly different from CTL. In contrast to the present data, Cook et al. reported lower ventilatory measures in ME/CFS at VAT ($\dot{\text{V}}$e, respiratory rate, and ventilatory equivalents of $\dot{\text{V}}$O_2_ and $\dot{\text{V}}$CO_2_) in their total sample, with some differences persisting in their matched-pairs sample. The higher ventilatory measures in their total control sample were consistent with the 28% higher aerobic capacity in their control group compared to ME/CFS. Additionally, in the present study, perception of effort (RPE 6–20 scale) at VAT was significantly higher in ME/CFS (11.6) compared to CTL (10.5) during CPET-1. Discussed elsewhere as well, this finding indicates that the subjective assessment of effort in ME/CFS is higher at the same metabolic transition point compared to controls [102]. When applied to daily living, activities that approach the intensity of VAT energy production will be perceived as relatively more effortful for ME/CFS. In conjunction with an overall lower work output at VAT, a higher RPE at VAT indicates that ME/CFS perceives work to be harder even when doing less work compared to controls. Despite being matched for $\dot{\text{V}}$O_2_ peak, the significant differences observed in $\dot{\text{V}}$O_2_ at VAT for the total sample persisted in the matched pairs as well.

Possible reasons for differences in ventilatory measures reported by Cook et al. may be due to participant screening and selection. Overall, the ME/CFS and control participants in the Cook et al. study had an 11% and 24% higher baseline peak $\dot{\text{V}}$O_2_ (ml^.^kg^.−1^min^−1^), respectively, compared to participants in the present study. Likewise, for their matched-pairs, baseline peak $\dot{\text{V}}$O_2_ for ME/CFS and controls was 14% higher than the matched-pair sample in the present study. It is also possible that differences are simply a reflection of the heterogeneity inherent in the ME/CFS population. However, for the controls, participants in the Cook et al. study were substantially more aerobically fit in contrast to the controls in the present study. This is not due to differences in age between the two samples as controls in Cook et al. were very similar in age (42.5 y ± 14.0) to the present study (42.8 y ± 13.4), but more likely associated with screening and assessment of chronic physical activity level for controls.

### Post-exertional measures at VAT

Not surprisingly, a deterioration in work, gas exchange and hemodynamic measures at VAT for ME/CFS in both the total and matched-pairs samples, with no change in perception of effort, elucidates the unique and persistent post-exertional response described previously [19, 25]. In contrast, there was an absence of change in CPET measures at VAT for CTL, with the overall stability of these measures indicated by the higher ICCs compared to ME/CFS (Table 6). Exerting below the VAT level of oxygen consumption proves to be a critical practice to minimize the impact of PEM and is a recommended strategy for ME/CFS, as evidenced by the data at VAT [103].

### Derived cardiac and ventilatory performance variables

Measures of cardiac performance were consistently lower in ME/CFS than CTL in both the total and matched-pairs samples despite meeting maximum effort criteria. In contrast during CPET-2, a lower CTI_peak_ indicated chronotropic incompetence in both the total and matched-pairs ME/CFS. This is consistent with previous reports of physical [27, 52, 92] as well as orthostatic stressors [104] attenuating the heart rate response during exertion, and adding to the myriad contributors of PEM in ME/CFS [1].

Cardiac performance measures at VAT ($\dot{\text{V}}$O_2_, Work, HR) were expressed as a percentage of peak values. While consistently lower in ME/CFS compared to controls, significant differences between phenotypes were most evident in the matched-pairs, especially during CPET-2. This is consistent with Davenport et al. [5] who reported that a significant decline in workload at VAT distinguishes the post-exertional response in ME/CFS. Lastly, OUES, the ventilatory requirement for a given VO_2_ and an index of cardiopulmonary functional reserve, was consistently lower in ME/CFS in the total and matched pairs groups. Similar findings were reported by Cook et al. [30] in their total sample but not in their matched pairs. Discussed previously, the differences in subject matching protocols between the two studies may account for different results regarding OUES in matched pairs.

### ICC

ICCs for CPET measures at peak exercise (Table 5) showed ‘good’ to ‘excellent’ consistency in 14 of 20 measures for ME/CFS and 15 of 20 measures for CTL in the total sample, and 14 of 20 measures for ME/CFS and 16 of 20 measures for CTL in the matched-pairs. Lindheimer et al. [105] reported ICCs for seven CPET measures from a 2-day CPET on a small sample of Gulf War Illness (GWI) veterans and controls to identify the smallest real difference to determine if declines on test 2 were clinically relevant and consistent with that of the GWI population [5, 21, 105, 106]. ICCs for work, respiratory rate, tidal volume, $\dot{\text{V}}$O_2_, $\dot{\text{V}}$CO_2_, and RPE were comparable to those reported for our total sample. ICCs for heart rate at peak $\dot{\text{V}}$O_2_ were lower in the present study (0.79 for ME/CFS, 0.79 for CTL) compared to Lindheimer et al. [105] (0.91 GWI, 0.85 controls). A possible explanation for the difference is that ME/CFS who did not meet maximum effort criteria for CPET-2 were included in our analysis, whereas Lindheimer et al. excluded GWI who did not meet maximum effort criteria for test 2. Consequently, peak HR was more variable between CPETs in the present study. The rationale to include ME/CFS participants who did not meet maximum effort heart rate and/or RER criteria but met the RPE criteria was driven by the characteristic symptoms of exertion intolerance and PEM that define ME/CFS [1]. Post-exertional responses to CPET-1 consist of a variety of symptoms, which may include chronotropic incompetence. Recognizing that as a manifestation of PEM, we included ME/CFS participants who met maximum criteria for CPET-1, but not CPET-2, only if perception of effort also indicated a very strong/maximum effort (RPE =  ≥ 17).

Like Lindheimer et al. [105], ICCs at VAT were generally lower and more variable than ICCs at peak $\dot{\text{V}}$O_2_. Further, ICCs at VAT were similar between both studies except, again, for heart rate, where ICCs were lower in this study, likely due to the differences in sample size and participant inclusion. Ultimately, Lindheimer et al. concluded that PEM-related changes on test 2 should be considered in the context of CPET measurement variability that is characteristic of GWI, and by extension, this approach should be applied to ME/CFS despite not having measured subjects with ME/CFS. Objective impairment standards are established based on functional deficits compared to healthy individuals, not relative to those with a disease that causes a functional deficit [67]. Thus, comparing a post-exertional decrement in CPET measures within the context of the disease-related variability does not account for the disease-related decrement in function compared to healthy individuals. It is the latter that is relevant and essential to establish in those with ME/CFS when objectively assessing functional impairment and ability to tolerate activities of daily living and job demands. The stability of CPET measures has long been established for a variety of healthy and diseased populations [94, 95, 107–110], thus, for one with ME/CFS, a change in CPET measures during a two-day CPET protocol should be considered within the context of the well-established normal variability.

### Impairment status

Overall, the post-exertional (CPET-2) effects on impairment in ME/CFS were similar to those reported by van Campen et al. [111] in patients categorized based on illness severity. As shown by van Campen et al., patients with clinical symptoms of mild, moderate, and severe ME/CFS all experienced reductions in energy production at peak effort and VAT during test 2, with the largest decline in the severely-ill patient group. The present study reinforces those findings, but also demonstrates the stability of impairment status across CPETs and thus the lack of post-exertional impact in sedentary controls.

### Limitations

Although the largest study to date of ME/CFS completing a 2-d CPET protocol, the primary limitation of this study is, in fact, the sample size. The disparate findings of this study compared to the larger 1-d CPET study of ME/CFS [30] suggests that a larger sample size is needed to more fully characterize the post-exertional impact of exercise on ME/CFS. Results of the present study notwithstanding, the effect sizes for many highly significant differences between phenotypes and between CPETs were small to moderate, possibly qualifying the practical application of the findings. However, given that post-exertional declines in oxygen consumption, ventilation, hemodynamic measures, and work, particularly at ventilatory/anaerobic threshold, are consistent with previous reports of 2-d CPET [5, 19, 22–26, 28, 92, 96, 112, 113], the stability of the present results only begin to describe the true magnitude of post-exertional effects for those with ME/CFS.

As with any study using open circuit spirometry to measure oxygen consumption via gas exchange, the ability to accurately differentiate central (cardiac) contributions to blood flow and peripheral (skeletal muscle, etc.) extraction of oxygen for energy metabolism is limited without direct measures of blood gases. For example, a 2-d CPET study of healthy, active males with an intervention between CPETs of intense eccentric exercise (100 squats), demonstrated that the gas exchange threshold (VAT) and the lactate threshold determined by sequential sampling of mixed venous blood, were effectively disengaged following the exercise regimen. This study suggested that minute ventilation to determine VAT may be altered by neurogenic stimuli following intense eccentric exercise [114]. While there was no between-CPET intervention in the present study, this does illustrate the importance of controlling participant behavior before and during the 2-d CPET protocol. This is to assure, as best is possible, that participants begin the protocol in a rested baseline state and do not intervene between CPETs with any modalities (e.g., massage, additional medications, etc.) to alter the true post-exertional response to CPET-1. In this study, specific instructions were given regarding preparation for and conduct during the 2-d CPET protocol to enable a valid comparison of results from each CPET [39]. However, a direct measure of blood gases obtained during an invasive CPET [115–117] would provide additional information regarding peripheral oxygen extraction. Although, an invasive CPET would still not clarify the issue brought forth earlier regarding ANS signaling of blood flow and oxygen delivery. Simultaneous measurement during exercise of peripheral blood flow by Doppler ultrasound or at least ankle-brachial index or other means, would help to understand the effects of ANS dysfunction on microcirculatory flow [118].

The use of a cycle ergometer as an exercise modality for CPET when testing ME/CFS is preferred over a treadmill for several reasons. Risk of losing balance and falling is greater on a treadmill particularly at high exertion, and quantification and reproduction of workload is more accurate on a cycle – which is critical for validity of a 2-test protocol. However, oxygen consumption at maximum effort on a treadmill may be 10–15% higher compared to measurement on a cycle, stepper, rower or ski ergometer [119], due largely to familiarity of walking compared to other activities. Still, the benefits of using a cycle ergometer for testing ME/CFS is favored for safety and reasons explained above, as evidenced by use in other exercise studies of ME/CFS [5, 19, 22–26, 28, 30, 92, 96, 112, 113, 120–122].

This multi-site study afforded participant selection from urban, suburban, and rural locales. While participants included representation from multiple races and ethnicities, participants with ME/CFS were 90% + Caucasian. The proportion of females in this study with ME/CFS was similar to that reported elsewhere [3], although sex differences were not evaluated in the analysis of the present data [123]. In addition, ME/CFS participants were primarily of mild to moderate illness severity, indicated by the Bell Activity Scale [35]. As such, the results of this study may not be generalizable to all races [124], ethnicities, or those severely ill with ME/CFS.

## Treatment considerations

In addition to the present study, disrupted post-exertional hemodynamic, ventilatory, metabolic function, and symptom complex are ubiquitous findings in studies of ME/CFS. Central to these findings is a post-exertion reduction in energy metabolism and work output. The Fick Principle [125] reminds that oxygen utilization for energy production is wholly dependent on blood flow and tissue oxygen extraction. This is true for any tissue (e.g., brain, skeletal muscle, liver, etc.). For cardiac tissue, this relationship is described as:

$$\mathop Q\limits^{.} = \mathop V\limits^{.}{\text{O}}_{{2}} /({\text{C}}_{{\text{a}}} - {\text{C}}_{{\text{v}}} )$$

Where, $\dot{\text{Q}}$ is cardiac output, $\dot{\text{V}}$O_2_ is oxygen consumption, C_a_ is oxygen content in arterial blood, C_v_ is oxygen content in mixed venous blood, and (C_a_-C_v_) represents the volume of oxygen extracted by tissue from blood for energy metabolism. Rearranging this equation to solve for $\dot{\text{V}}$O_2_ becomes:

$$\mathop V\limits^{.}{\text{O}}_{{2}} = \mathop Q\limits^{.} \, \times \,({\text{C}}_{{\text{a}}} - {\text{C}}_{{\text{v}}} )$$

Using this version of the formula, it is easy to see that the volume of oxygen consumed for energy metabolism ($\dot{\text{V}}$O_2_) is a function of the volume of blood pumped ($\dot{\text{Q}}$) to the tissue (e.g., skeletal muscle), and the volume of oxygen extracted by the target tissue (C_a_-C_v_). To measure $\dot{\text{V}}$O_2_ using the non-invasive analysis of expired gases requires the knowledge of minute ventilation (volume of air that moves through the lungs per minute). Thus, it is clear that delivery of oxygen to tissue for energy metabolism relies on blood flow and ventilatory function. At rest, and especially during exercise, the regulation of blood flow and ventilation depend on coordinated feedback from the body to the brain stem regarding tissue oxygen status. Based on this information, commands are elicited to up- or down-regulate blood flow ($\dot{\text{Q}}$), as necessary, to provide oxygen for energy metabolism. Under the auspices of the ANS, this tightly regulated messaging normally happens seamlessly and involuntarily.

With this understanding and given the disordered hemodynamic and ventilatory responses to exertion in ME/CFS, it is plausible to consider treatment approaches that may help to re-regulate ANS signaling for improved oxygen delivery to the heart, skeletal muscle, gut, brain, and other tissues impacted by ME/CFS. Unfortunately, relatively little attention and resources have been directed toward non-pharmacological approaches to reduce symptoms of ME/CFS. Despite compelling evidence of ANS dysfunction in ME/CFS [104, 126–130], there are no randomized controlled trials to assess treatment approaches. Here, we offer strategies proffered variously by many with ME/CFS, trial and error, practice, and some clinical evidence that center on non-pharmacological opportunities to possibly mitigate pain and inflammation, and enhance blood flow, oxygen delivery, and/or tissue oxygenation.

Return of blood to the heart (venous return) may be assisted by use of compression garments (shorts, tights, stockings, shirt, sleeve) which are effective to aid recovery by improved blood flow in athletes [131–133] and in ME/CFS for those who experience orthostatic intolerance [134]. Massage may help to reduce depression, anxiety, stress, and perception of fatigue, and enhance overall mood and relaxation to indirectly promote blood flow, although it may not alter blood flow directly [135].

Core stability exercises to maintain or improve effectiveness of muscles of the trunk and hips to support correct spinal alignment can help to improve circulation and oxygen delivery [136, 137]. These can be done while lying down so as not to exacerbate orthostatic symptoms. Most important when doing core stability exercises is to maintain correct spinal alignment during the exercise, more than duration or repetitions of the exercise. The focus of core stability exercises is to improve the coordination of these muscle groups and intra-abdominal pressure regulation by the central nervous system [138]. When first learning these exercises, working with one experienced and knowledgeable in teaching core or neuromuscular stabilization exercises , such as a physical therapist, athletic trainer, or strength/conditioning coach, would be advised to provide guidance and feedback. As with all exercise, core stability exercises should be performed within the limitations of exertion tolerance, discussed below, so as not to exacerbate post exertion symptoms. Recognize, however, that local muscle sensitivity may arise when first beginning these types of activities which is common when first exerting muscles to perform an unfamiliar task.

Vagus nerve stimulation (VNS) is an FDA-approved treatment for pharmacoresistant depression and epilepsy, producing clinically meaningful results. Implantation of a VNS device requires a surgical procedure and is not without risk. More recent attention has focused on transcutaneous vagus nerve stimulation (tVNS) using an external device to stimulate the vagus nerve at either the ear to access the auricular branch of the vagus nerve or the neck to target the cervical branch of the vagus nerve. More is known about appropriate treatment protocols using tVNS for depression and epilepsy, although studies have also examined the use of tVNS for management of pain, migraine, tinnitus, and cognitive dysfunction, among other maladies [139]. For ME/CFS, preliminary findings suggest some efficacy of tVNS to reduce sympathetic stimulation of the heart, indicated by improved heart rate variability, but that ANS response varied depending on sex and tVNS stimulation parameters [140]. While more work is warranted to understand optimal treatment protocols, long-term effects, and other possible applications, substantial evidence indicates that tVNS can help to rebalance parasympathetic/sympathetic tone of the intrinsic cardiac nervous system to reduce heart rate [141].

Cryotherapy or cold therapy is a therapeutic modality to decrease pain, reduce chronic and acute inflammation, and to aid recovery in athletes, among other applications. Cold exposure of the whole body stimulates ANS responses to increase core temperature toward normal body temperature by redirecting blood flow away from the skin and toward the heart and viscera. In doing so, a very brief, intensely cold whole-body exposure is believed to provoke ANS re-regulation toward homeostasis or stable equilibrium, and thereby improve symptoms of ME/CFS associated with cardiovascular autonomic dysregulation [77]. Coupling brief whole-body cryotherapy with static stretching improved symptoms of ME/CFS related to fatigue, sleep, and cognitive function [142, 143]. More work is needed to better elucidate the mechanisms involved in the effectiveness of whole-body cryotherapy, although preliminary data suggests a promising approach to consider for symptom mitigation.

Evidence of structural and functional abnormalities in the brain of some with ME/CFS may be related to accumulation of toxins associated with glymphatic dysfunction [144]. Similarly, the primary respiratory mechanism, typically dysregulated in those with ME/CFS, is suggested to be synchronous with the rhythmic pulsation of lymphatic drainage from the brain and spinal cord, or neuraxis, induced by sympathetic nervous system activity. Consequently, impaired cranial rhythmic impulse could lead to respiratory dysfunction, chronic fatigue, and other symptoms of ME/CFS [145]. Some evidence indicates that a specific manual lymphatic drainage intervention may reduce fatigue symptoms in long COVID and chronic venous insufficiency [146] which share many symptoms with ME/CFS.

Although not fully understood, photobiomodulation, also known as low-level laser therapy (LLLT) or red-light therapy, has been in existence for more than a half century. It is known to affect mitochondrial function by altering cytochrome c oxidase which is particularly able to absorb light in the near-infrared region, increasing electron transport activity and ultimately adenosine triphosphate production[147, 148]. More commonly used now in sport medicine and orthopedic rehabilitative settings to enhance recovery and repair, LLLT has been reported to decrease soreness, inflammatory markers, lactic acid, and oxidative stress, and enhance glucose uptake to support aerobic metabolism [149–151].

An additional approach to modulate systemic inflammation involves selection and timing of body fuels. Nutritional considerations to reduce inflammation include an anti-inflammatory diet, reduced consumption of refined sugar, a gluten-free diet, or intermittent fasting [152]. A nutritional approach to ‘feed’ the gut microbiome can help with energy balance, glycemic control, and inflammation [153].

It has long been known, but possibly underappreciated, that the integrity of fascia in the maintenance of muscle tension and interstitial pressure is integral to healthy muscle function and force production, independent of muscle fatigue [154]. As such, fasciotomy, the common approach to relieve the pain of muscle compartment syndrome by cutting the surrounding fascia, unfortunately also reduces muscle force output by 50% or more [155]. However, fascia that is unusually restricted or foreshortened may increase neural tension and contribute to pain, altering muscle recruitment patterns, reducing muscle force production and intra-neural blood flow, and releasing inflammatory factors [156]. In those with ME/CFS, longitudinal strain to the nerves and soft tissue of the lower limb increased pain and many symptoms of PEM, including difficulty concentrating. Prolonged sitting, reclining bed rest, or driving with arms outstretched and right leg extended are examples of activities that could contribute to increased mechanical tension to the nervous system [157].

Efforts to reduce fascial restriction and mechanical tension using myofascial release therapy effectively decreased pain and improved range of motion and functionality in women following breast cancer surgery [158]. Both the subcutaneous and subserous fascial planes slide independently but fuse at specific locations, particularly in the area of the pelvis, abdominal wall, and aperture of the thorax [159], suggesting that impingement of fascia in one area can ‘tug’ on an adjacent or even distal area provoking symptoms that seem unrelated to the point of restriction. For example, a head injury may later contribute to pain lower in the body due to adhesions that ‘pull’ on the longitudinal axis of the fascia causing pain elsewhere. Approaches that may bring relief by way of liberating fascia include appropriate physical therapy, body work, breathing exercises, gentle stretches, stress reduction, acupuncture, foam rolling, FasciaBlaster®, heating pad or hot water bottle, and/or nutritional support for fascia. In addition to circumstances described above involving prolonged stationary positions, those who have experienced injury, surgery, or some type of bodily trauma that could cause fascial adhesions may want to consider these approaches.

Blood flow restriction training (BFR) involves restriction of blood flow to arms or legs to trap blood in the local musculature during low-intensity resistance exercise, eliciting a strong hemodynamic response. It has been reported to increase strength and muscle size in healthy adults and reduce characteristics evident in chronic heart failure including muscle atrophy, shortness of breath, fatigue, increased ventilation, and sympathetic stimulation [160]. Exercise with BFR may also promote more angiogenesis-related factors mRNA expression and improve vascular function [161]. Collectively, these findings suggest that BFR training may be of benefit to those with ME/CFS to mitigate muscle loss but also to improve functional performance. More work is warranted to better understand the mechanisms of action with BFR, as well as an appropriate protocol for use in ME/CFS to avoid possible dizziness that has been reported in some cases, but it appears to be a promising approach to reduce fatigue and improve muscle function.

Activity pacing is a goal-directed behavioral approach that involves decision-making and planning to effectively manage available energy resources to reduce fatigue and symptoms of PEM in those with ME/CFS [103]. It is an approach that has gained acceptance, not only for ME/CFS, but for other disabling conditions, including long COVID. The goal of pacing, as opposed to graded exercise therapy, is symptom reduction to improve well-being and overall function through self-regulatory behavior [162]. Effective pacing can reduce fatigue, psychological distress, depression, and improve overall physical function [163]. Activity pacing relies on basic journaling of symptoms and activities to provide a ‘look-back’ in instances where PEM symptoms emerge to understand possible triggers of symptom exacerbation and inform future pacing-related decisions. Additionally, it is helpful to have an objective indicator of exertional threshold to provide on-going feedback. Based on data from the present study, it is evident that exertion above VAT provokes abnormal hemodynamic and ventilatory responses. The VAT level of energy production can be ascertained during a submaximal cardiopulmonary exercise test. When heart rate that corresponds to VAT (HR@VAT) is known, use of a simple heart rate monitor with an alarm set to 10 bpm below heart rate at VAT provides objective, auditory feedback to reduce exertion below threshold to avoid symptoms of PEM.

When heart rate at VAT is not known, RPE may provide an estimate of VAT. An upper exertion limit RPE of 10–12 (for a 6–20 scale) or RPE of 2–3 (for a 1–10 scale) are consistent with ranges in perceived exertion from ‘*light and easy – non-taxing, very gentle and easy to maintain a conversation – could continue for hours*’ to ‘*comfortable pace – able to maintain a conversation without getting out of breath*’. Pacing should begin with a conservative estimate of exertional threshold (e.g., 10/20 or 2/10, or less if indicated) while using journal entries from the previous 1 to 2 days to assess if the exertional limit is effectively mitigating symptom exacerbation. If not, then the exertional limit should be lowered, recognizing also that the cyclic nature of symptoms in ME/CFS may alter exertional tolerance levels at times. Another exertional metric to begin pacing when heart rate at VAT is not known can be estimated by adding 15 bpm to resting heart rate (RHR + 15). Resting heart rate should be measured after 5 min of seated or supine rest in a quiet environment. Preliminary data from the Workwell Foundation suggests that RHR + 15 is a useful metric to guide pacing when HR@VAT is not known, with more information about pacing at www.workwellfoundation.org. A positive and protracted adaptation to, and familiarity with pacing is necessary before venturing to raise the exertional tolerance level. Effective pacing over time may allow for a very gradual escalation of physical and cognitive activities [163]. Emergence of post-exertional symptoms is always an indicator that prior exertional levels exceeded exertional tolerance.

One indicator of successful symptom mitigation over time may be the measure of heart rate variability (HRV). This is the measure of variability in time between each heartbeat and is an indirect indicator of ANS tone. Increasing evidence suggests that the ANS regulates the inflammatory response. A decrease in HRV indicates an abnormal predominance of sympathetic activity and has been observed in patients with ME/CFS [164, 165], fibromyalgia pain [166], post-traumatic stress disorder [167], type 2 diabetes [168], and as a general indicator of stress and health [169]. Neuroimaging studies indicate a relationship between HRV and regional cerebral blood flow, suggesting that the interpretation of external phenomena as threat or negative (ie., ‘fight or flight’ response) can alter higher brain messaging to the brainstem and subsequent ANS signaling to the heart [169]. Additionally, one’s interpretation of psychosocial stress can be quantified by measuring cortisol release, which is closely associated with HRV, as an indicator of the physiological response to the stress [170]. In this way, HRV can be used to track changes in autonomic tone over time and as an indicator of productive symptom management in ME/CFS.

Breathing and circulation are related. Impaired respiratory function is universal in ME/CFS and so must be addressed to improve circulation of blood with oxygen to tissues for energy metabolism and circulation of lymph to remove metabolic byproducts. Although breathing is an involuntary process, intervention using voluntary slow or diaphragmatic breathing can effectively improve vagally-mediated HRV with only a little training [171, 172]. Slow breathing can reduce blood pressure in low-risk hypertensive and prehypertensive patients [173], hypertensive diabetics [174], and improve respiratory function in chronic obstructive pulmonary disease, reduce stress, anxiety, constipation, migraine, and other ANS-associated maladies. Among other effects, the diaphragm also influences postural stability, elimination, birthing, metabolic balance, cardiovascular and lymphatic systems [175]. Given the broad reaching impact of improved breathing mechanics, this low-risk, low-cost, non-pharmacological approach to symptom mitigation should be a first-line approach.

Finally, ANS function is intimately associated with neurophysiological responses, including ‘fight or flight’, immune activation, pain sensitivity, and many other stress-related impacts. Understanding this is at the core of integrative approaches to help move away from ‘sickness’ and toward ‘health’. There is increasing evidence of the efficacy of some types of ‘mindfulness training’ to transcend the state of ‘ill’ and move toward a state of ‘well’ with regard to many diseases [176]. Recent work demonstrated the efficacy of meditation as an adjuvant therapy to alter bloodborne factors and resiliency to viral infection in the treatment of COVID-19 [177] and has been reviewed elsewhere with promise for long COVID and ME/CFS [178]. A multi-symptom disease such as ME/CFS is often addressed with a poly-pharmaceutical approach to symptom management. Consideration by both patient and physician of the approaches discussed herein could reduce the efforts to pharmacologically control one’s physiology through the understanding that many, if not all symptoms of ME/CFS implicate ANS dysfunction and may be positively influenced with non-pharmacological approaches. The body-brain axis is proving to be highly influential in control and regulation of energy metabolism[179].

## Conclusions

Baseline (CPET-1) aerobic capacity was similar and low in these groups of ME/CFS and inactive controls, although it was only ME/CFS that experienced a notable reduction in peak $\dot{\text{V}}$O_2_ on CPET-2. However, it is the relevance of a lower baseline gas exchange threshold (VAT) that underscores the challenge of those with ME/CFS to accomplish daily activities without exceeding the VAT level of energy production and exacerbating illness symptoms. In addition to an array of illness symptoms that comprise the post-exertional response in ME/CFS, the reduction in VAT effectively shrinks the ‘energy envelope’ further reducing tolerance of energy-demanding activities. These results implicate oxygen consumption at VAT, or a shift in energy production increasingly to anaerobic processes, to be central in the metabolic dysfunction of ME/CFS [180]. Similarly, these same findings in ME/CFS extended to the case–control pairs matched for sex, age, and peak $\dot{\text{V}}$O_2_ indicating that a low aerobic capacity does not explain the deleterious impact of exertion intolerance in ME/CFS. Ventilatory dysfunction was another prominent feature of ME/CFS in both the total group and matched-pairs cohort, with ventilatory inefficiency emerging particularly at the VAT level of exertion. Applying measures of peak $\dot{\text{V}}$O_2_, $\dot{\text{V}}$O_2_@VAT, and $\dot{\text{V}}$ /$\dot{\text{V}}$CO_2_ to established impairment standards revealed that for ME/CFS; 1) severity of impairment was worse based on results from CPET-2 compared to CPET-1, 2) severity of impairment was worse in ME/CFS compared to controls, even in ME/CFS matched for sex, age, and peak $\dot{\text{V}}$O_2_, and 3) an alarming proportion of inactive control subjects met impairment standards based on these physiological indices. Because post-exertional malaise is a hallmark symptom of ME/CFS, assessing severity of impairment must account for the diminished energy producing capacity due to PEM.

Considering the post-exertional declines unique to ME/CFS for various indices of cardiovascular performance and ventilatory function logically incriminates the role of the autonomic nervous system as a preeminent factor contributing to metabolic dysfunction in ME/CFS. Dysregulated blood flow and pulmonary function that may reduce oxygen delivery to tissues for energy metabolism will force a premature and disproportionate increase in anaerobic, rate-limiting, fatigue-inducing metabolism that is characteristic of ME/CFS. The many and often disparate-appearing symptoms of ME/CFS may be explained by understanding the role of the ANS in the relationship between the body and brain [179]. Coupled with findings of endothelial dysfunction [181], immune dysfunction, and other abnormalities [182] regulated directly or indirectly by the ANS, treatment approaches to reclaim healthy ANS function seem in order.

Baseline functional capacity and post-exertional physiological responses in ME/CFS remain to be fully understood. Although the largest study to date, future studies with more participants are needed to accurately describe the magnitude of exertion intolerance in ME/CFS and to delineate possible subsets of CPET responses in ME/CFS. The relationships between peak oxygen consumption, impairment severity, and patient responses on the MOS SF-36 questionnaire [183] suggests that a first-line assessment by physicians might begin with completion of the MOS SF-36 [184]. Finally, long overdue evidence-based guidance must be provided to clinicians, therapists, and those with ME/CFS for activity management and safe, metric-guided progression of exertion to improve function and exertion tolerance. This calls for, (1) a study with a robust sample size to validate the present findings and to more fully characterize phenotypes of ANS dysfunction in ME/CFS [184], and (2) clinical trials to devise effective precision treatment approaches to help people with ME/CFS.

## Data Availability

The datasets generated and/or analyzed during the current study are available in the mapMECFS repository (www.mapmecfs.org).

## Abbreviations

ANS: Autonomic nervous system
BFR: Blood flow restriction training
BMI: Body mass index
BP: Blood pressure
BPM: Beats per minute
BSA: Body surface area
CPET: Cardiopulmonary exercise test
CRC: Collaborative Research Center
CTI: Chronotropic index
CTL: Healthy controls
DBP: Diastolic blood pressure
ECG: Electrocardiogram
GWI: Gulf War Illness
HEENT: Head, eyes, ears, nose, and throat
HR: Heart rate
HRR: Heart rate reserve
HRV: Heart rate variability
ICC: Intraclass correlation
IOM: Institute of Medicine
LLLT: Low-level laser therapy
ME/CFS: Myalgic Encephalomyelitis/Chronic Fatigue Syndrome
OUES: Oxygen uptake efficiency slope
PEM: Post exertional malaise
P_ET_CO_2_: End tidal carbon dioxide pressure
P_ET_O_2_: End tidal oxygen pressure
PP: Pulse pressure
$$Q$$: Blood flow
RER: Respiratory exchange ratio
RPE: Rating of perceived exertion
RPP: Rate pressure product
RR: Respiratory rate
SBP: Systolic blood pressure
TPR: Total peripheral resistance
TTL: Time to level
tVNS: Transcutaneous vagus nerve stimulation
VAT: Ventilatory/anaerobic threshold
$\dot{\text{V}}$CO_2_: Volume of carbon dioxide production
VCP: Ventilatory compensation point
Ve: Minute ventilation volume
$\dot{\text{VC}}$O_2_: Volume of carbon dioxide production
$\dot{\text{V}}$O_2_: Volume of oxygen consumption
$\dot{\text{V}}$O_2_peak: Volume of oxygen consumption at peak effort
VNS: Vagus nerve stimulation
$$\dot{\text{V}}$$_T_: Tidal volume
